# Supplementary material for: Open-source cell culture automation system with integrated cell counting for passaging microplate cultures
Source: PNAS Nexus. 2025 Dec 30;4(12):pgaf385. doi: 10.1093/pnasnexus/pgaf385 (PMC12750449; doi:10.1093/pnasnexus/pgaf385)
Supplement: pgaf385_Supplementary_Data [file pgaf385_supplementary_data.pdf]

ACCS: Open-source cell culture automation system with integrated cell counting for passaging microplate cultures

Supplement A.
Supplemental Materials and Methods

Contents

ACCS passaging protocol
Flow cytometer settings
Supplemental figures

ACCS passaging protocol

This section expands on the steps of the automated passaging process in more detail than could be accommodated in the main article.

Growth media and dissociation reagent are warmed to 37°C prior to loading on the robot; any other reagents are loaded at room temperature. HEPES is used in the media as a precaution to ensure stable pH since the plates spend more time out of the CO_2 incubator atmosphere than they typically would when passaging by hand. See the Reagents and Cultures subsection of the main article for a full enumeration of the reagents used for ACCS protocols.

Harvesting of cells from the source plate is carried out columnwise with the 8-channel pipette. The harvesting phase consists of aspirating and discarding the old media, gently washing the adhered cells with 100 uL of DPBS, adding 60 µL of dissociation reagent, incubating for approximately 8 minutes, quenching with 200 µL of growth media to neutralize trypsin, then mixing vigorously to dissociate the cells into singlets as effectively as possible. From the start of the protocol, the block heater is held at a setpoint of 41°C which we have empirically found to maintain the bottom of the plate as close as practical to 37°C; after all wells have been quenched the block is allowed to coast back to room temperature.

Following the dissociation step, if seeding according to cell density targets, the CCI is used to measure the concentration of harvested cells in each well. The process consists of taking a 30 µL sample from a column using the 8-channel pipette, injecting the samples into the CCI, waiting for 1 minute for the cells to settle in the chambers, then running a CCI counting cycle. The CCI takes about 100 seconds to acquire the images and perform analysis for all 8

channels, then the channels are immediately flushed with DPBS. In the case of a "blind split", the CCI is not involved, no sample is taken for counting, and seeding volumes are calculated directly from dilution ratios specified by the user.

The cell concentration values returned by the CCI software are used to calculate the appropriate volume to transfer for each well. Seeding volumes are constrained to a range of 20 µL to 200 µL, dictated at the upper end by the amount of cell suspension that can be reliably recovered from the source well and at the lower end by the volume range rating of the pipette. If the calculated volume is outside this range, a warning is issued and the value is clipped to the top or bottom of the range as appropriate. Practically speaking we find that aliquots are generally reliable up to 180 µL, above which there is greater likelihood of air bubbles being drawn into the pipette tip.

Seeding of the output plates is carried out with the single-channel pipette. For each well, first the appropriate quantity of media for dilution is added to the output well, then the cell suspension in the source well is mixed once more to ensure homogeneity before finally transferring the desired number of cells to the output well.

When seeding a duplicate plate, each column of the first plate is seeded with the sum of the cells and media for both copies, then the 8-channel pipette is used to mix the contents and transfer a portion of the total mixture to the duplicate. The amount transferred is controlled by a user-specified ratio, typically between 1:3 and 1:1, allowing the generation of, for example, a stock plate seeded at 25k cells/well and an imaging plate seeded at 12.5k cells/well.

Throughout all steps, the use of pipette tips is managed in such a way as to avoid cross-contamination of samples. Because the liquid volumes for adding trypsin and media to the source plate during harvesting are not especially critical, these dispensing steps are accomplished with the pipette tips hovering above the wells so as to avoid contact with cells and allow a single set of tips to be used on a whole 4-column group. For all steps involving contact with cell suspension, tips are discarded and replaced between plate locations. During seeding of the output plate(s), any media needed for dilution is transferred first, then the same tip is reused for transferring cell suspension.

Flow cytometer settings

This section lists the settings we used on the BD FACSymphony A1 when using it as a high throughput cell counter for ACCS seeding performance tests.

Cytometer parameter	Value
Trigger logic	OR
Window extension	0.00 µs

High Throughput Sampler parameter	Value		Detector	Recording channels	Voltage	Trigger Threshold
Loader mode	Standard		FSC	FSC-H, FSC-W, FSC-A	350 V	30000
Sample volume	60 µL		SSC	SSC-H, SSC-W, SSC-A	225 V	
Injection rate	3.0 µL/s		BB515	BB515-H	210 V	1000
Mixing volume	75 µL		BB700	BB700-H	285 V	
Mixing speed	180 µL/s					
# of mixes	5					
Wash volume	800 µL					

Supplemental figures

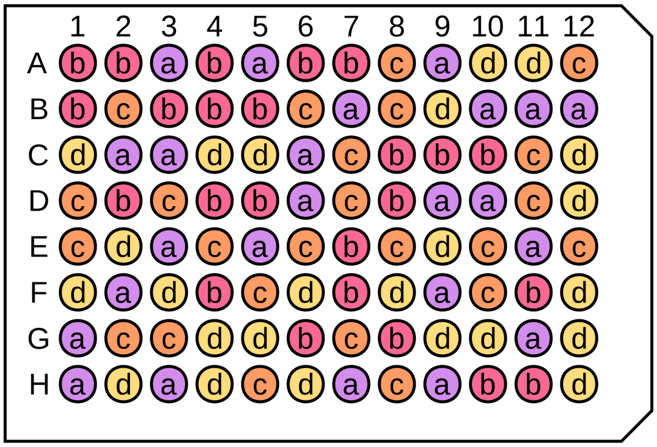

Supplemental Figure S1. Challenge Plate layout.

Four groups of 24 wells are randomly distributed on the plate. Each group is assigned a relative seeding density, ranging linearly from 50% for "a" wells to 100% for "d" wells.

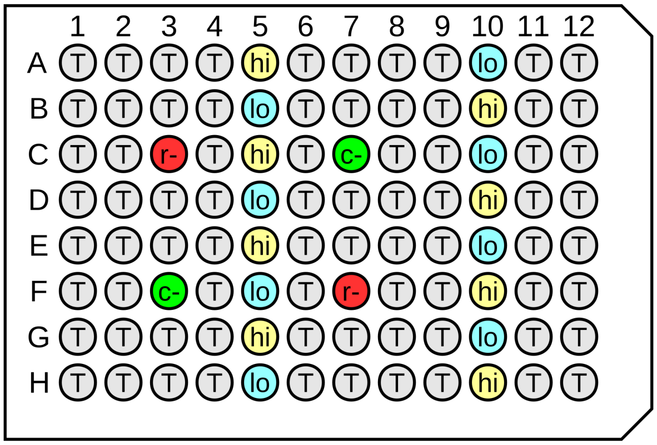

Supplemental Figure S2. CellTiter-Glo assay plate layout.

Wells marked "r-" and "c-" are "no reagent" and "no cells" negative controls, respectively. Wells marked "hi" and "lo" are seeded from prepared stocks with 20k and 10k cells, respectively. The 76 remaining wells marked "T" are the sample set for the test.

ACCS: Open-source cell culture automation system with integrated cell counting for passaging microplate cultures

Supplement B.
ACCS Integrator's Manual

- Introduction
- Hardware components
    - Liquid handler robot and accessories
    - Operator PC
    - Cell Counting Imager (CCI)
    - CCI cover
    - Tip waste bin (3-0390) and liner
    - Tilted heat block adapter (3-0271)
    - Tilted plate calibration tool (3-0938)
    - Reservoir riser (3-1482)
    - Inspection mirror and rear view mirror
    - Other tools and facilities
- OT-2 setup and modifications
    - Pipette modules
    - Side / top panels
    - Tip waste bin removal
    - Ethernet connection
    - Software environment
- Hardware installation
    - Deck layout
    - Installation in a biosafety cabinet
    - Power and electronic connections
- PC software setup
- Equipment list
    - Core off-the-shelf equipment
    - Tools
    - Custom components
    - Other
- Consumables
    - Lab plastics
    - Cleaning and disposal supplies
    - Reagents

Introduction

This document is designed to provide practical guidance to someone considering implementing ACCS for their own lab. It assumes a basic level of familiarity with hardware prototyping, computer software and wet lab practice.

Hardware components

Liquid handler robot and accessories

ACCS is built around an Opentrons OT-2 liquid handling robot with the following Opentrons accessory equipment:

- 8-channel P300 Gen2 pipette -- used for columnwise processing steps on the source plate (media removal, washing, dissociation) and for loading and flushing the CCI
- 1-channel P300 Gen2 pipette -- used for seeding destination plate
- Temperature Module Gen2 -- temperature-controlled heating/cooling block, used to incubate the source plate prior to and during trypsinization

Operator PC

The only strict hardware requirement is that a dedicated gigabit ethernet port must be available for the CCI camera.

We use a Windows 10 system in production. The ACCS and CCI software and their dependencies are cross-platform so in principle a Linux or macOS system could be used instead.

Note that the CCI software as-supplied is designed with the assumption of one CCI per PC, so some slight modifications would be required to operate multiple CCIs simultaneously.

Cell Counting Imager (CCI)

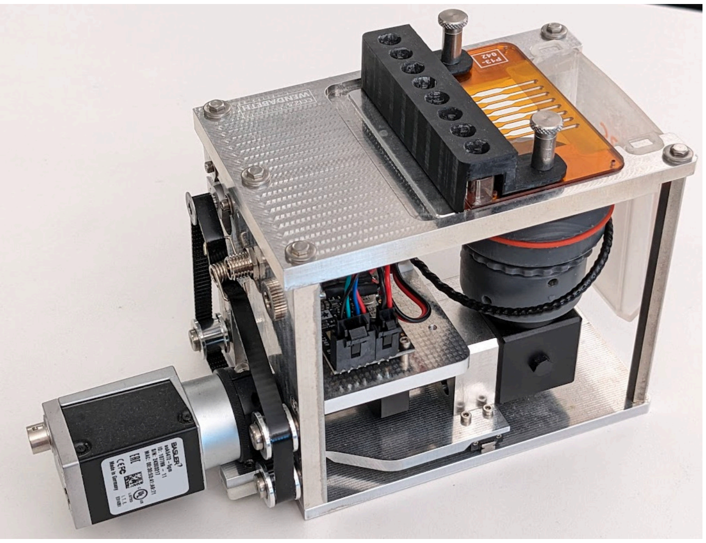

Refer to the separate *ACCS Cell Counting Imager Technical Manual* for information on the instrument itself, the flow cell, and support equipment and consumables related to CCI operation.

CCI cover

An improvised cover for the CCI can be made by cutting notches out of the corners of an Opentrons tipbox lid to clear the CCI fastener heads, as shown below.

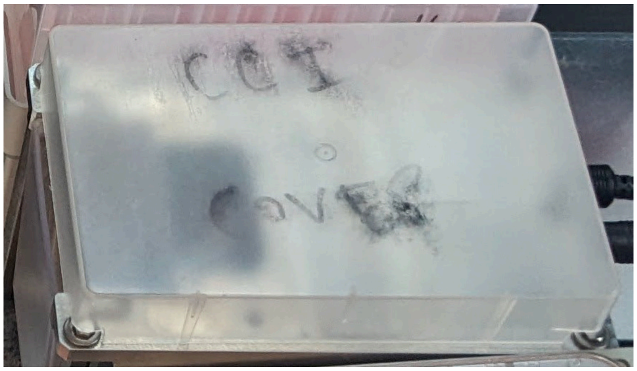

The CCI should be covered when spraying down the deck for disinfection or when the system is not in use, as the objective faces upward.

Tip waste bin (3-0390) and liner

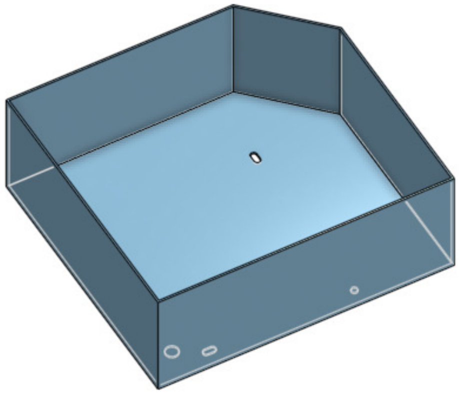

The tip waste bin replaces the small bin built into the OT-2. The tip waste bin is made of 3D printed PLA plastic. The material used for the bin is not critical.

The bin is used with a liner made by cutting a 19" x 23" biohazard bag (Heathrow Scientific HS10322) down to about 10" height. The bin liner is installed by pushing the bottom of the bag into the waste bin, then folding the excess material over the edges and securing with stainless steel binder clips (e.g. McMaster-Carr 12755T81) so that the overhanging material sits flat against the sides of the bin on the two long sides. **It is important to avoid bunching of material on the floor of the bin as much as possible** to reduce the tendency for tips to form tall piles in the drop area. It is also advisable to check on the bin at least once during a long protocol and break up tip pileups if they form.

The bin model includes fastener holes to secure it to the OT-2 deck but in practice we find it more practical to leave it loose so that it can be freely handled to install the liner. The bin is installed by simply placing it on the robot deck as shown:

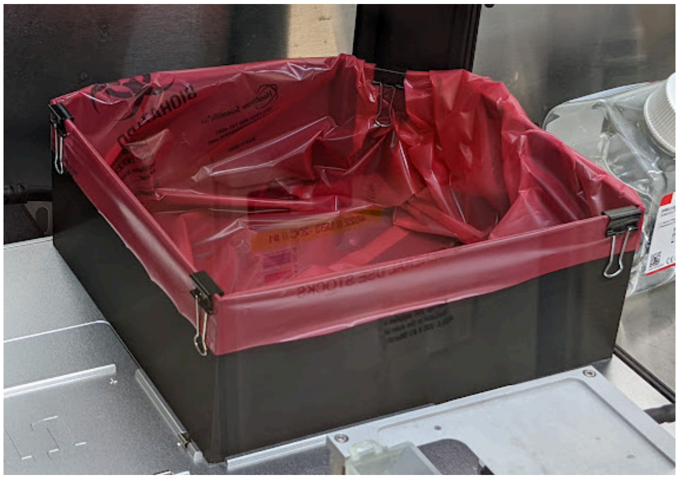

To accommodate the custom bin, the OT-2's built-in bin assembly must be removed as described in Tip waste bin removal.

An alternative single-use tip waste bin design made from laminated cardstock and featuring an inclined bottom is being considered to simplify setup and improve reliability.

Tilted heat block adapter (3-0271)

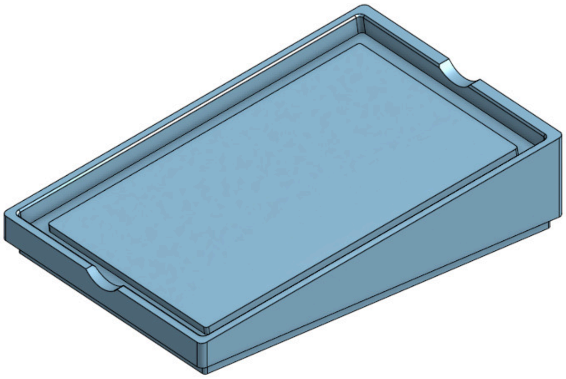

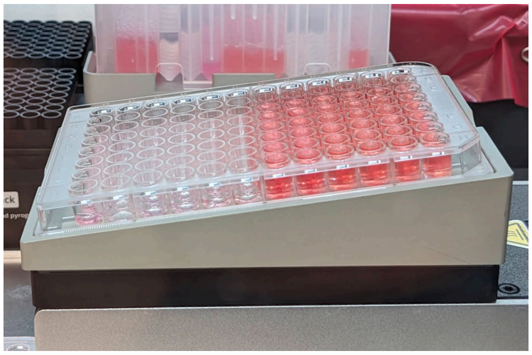

The tilted heat block adapter holds the source plate at an 8° incline while maintaining thermal contact between it and the Temperature Module. Tilting the plate is necessary in order to allow the pipettes to access the full volume of liquid in the wells. The narrow aspect ratio of the wells on a 96 well plate gives a generous allowance for tilt compared to plate types with wider wells which typically must be laid flat between pipetting steps to prevent the well bottoms being uncovered.

We find that a setpoint of 41°C on the Temperature Module results in peak well bottom temperatures just below 37°C and an acceptably uniform temperature (within 2°C) across the plate.

Because the Opentrons software does not currently support the concept of labware being mounted in a non-flat orientation, a special "tilted plate" labware definition file is used. A special tool to aid with labware position check / pipette calibration because the typical strategy of visually lining up the pipette tips to the rims of wells is not reliable.

The tilted heat block adapter is machined from 6061 aluminum and is bead-blasted followed by a Type II anodized finish. The bead blasting is not essential provided that sharp edges are broken by other means but anodizing is recommended for corrosion resistance.

The adapter is installed by simply placing it on the bare platen of the Temperature Module (ensure any included adapter is removed first, revealing the black surface) and is held in place by gravity. Finger notches are provided to make it easier to remove the microwell plate from the adapter.

The adapter is designed so that common plastic 96-well culture plates should drop in without resistance. Our testing was based on Thermo Scientific BioLite plates, cat. no. 130188.

Tilted plate calibration tool (3-0938)

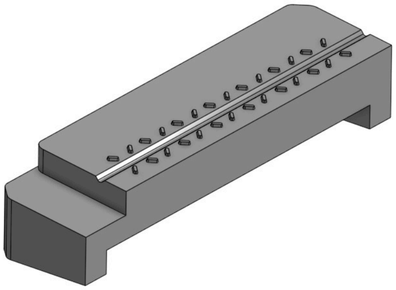

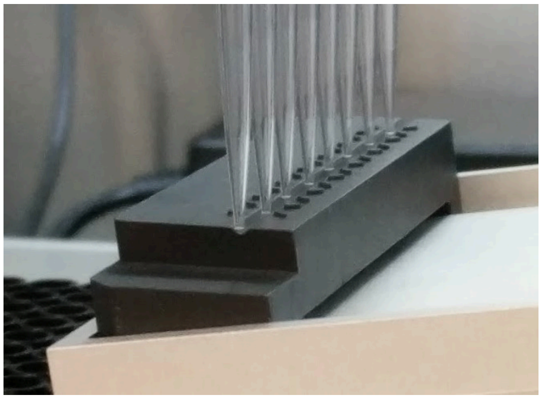

The tilted plate calibration tool is used to achieve non-contact alignment of the multichannel pipette with its calibration position during the Opentrons labware position check / calibration process. It was printed on a Formlabs Form 3 SLA printer using Formlabs Black Resin V4.

It is important that the tool sit in a consistent, stable and level position when placed on the tilted block; depending on the printing configuration, this may require sanding or machining the bottom of the tool. The height of the tool can be adjusted by shimming with Kapton tape or a similar material. The height should be set such that the tips just nearly reach (but never push against) the bottom of the source plate wells after calibration using the tool. A calibration position that is too high may result in under-aliquoting of cells and/or more frequent introduction of air bubbles during mixing. A calibration position that is too low may result in

some or all tips being blocked due to being held against the well bottoms, preventing proper aspiration.

Routine usage of the calibration tool is detailed in the *CCI Normalization Sample SOP*.

Reservoir riser (3-1482)

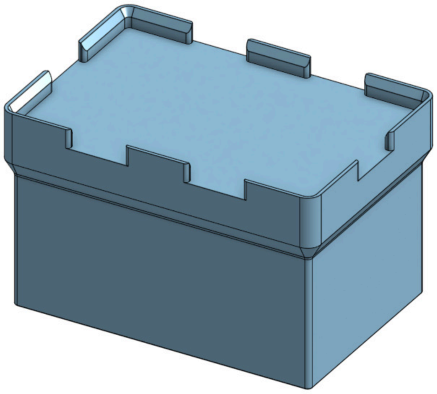

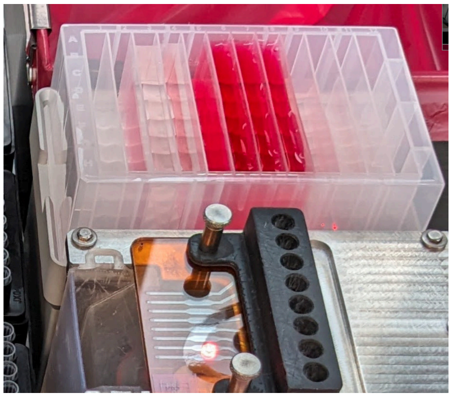

The reservoir riser allows drop-in installation of the reagent reservoir and elevates it by 76.5mm to make it easier for the operator to access it. It is machined from 6061 aluminum. Refer to finishing notes for the heat block adapter.

The reservoir riser was designed to address recurring issues related to mounting the reservoir directly on the deck. In particular, the design of the rails and spring clips on the OT-2's deck allows false placement of the reservoir such that it appears properly installed at a glance and feels secure, but is in fact out of position, leading to crashes and potentially catastrophic outcomes (such as the reservoir being picked up by the pipette and driven into the CCI). The reservoir riser is not an essential component but is recommended for these reasons. Although the solid metal construction is preferred for stability and hygiene reasons, 3D printing this part is a viable lower-cost alternative.

Inspection mirror and rear view mirror

Similarly to the reagent reservoir, the Opentrons tip boxes can inadvertently be installed out of position. This can be avoided by ensuring the rails are visible on all sides of each box, but this can be difficult when the OT-2 is installed in a recessed location such as inside a biosafety cabinet.

An inspection mirror with a telescopic handle (e.g. McMaster-Carr 1017T25) is useful for verifying that tipracks are seated properly. Additionally, we have installed a convex mirror

(United Pacific 43001) at the back of the robot to provide a rear view of the deck as shown below. The improvised mounting solution consists of a modified piece of aluminum angle attached to the robot using a set of small panel clamps (AES Industries AES-20410). The mirror is positioned such that the bottoms of the rearmost tipboxes are visible from the operator's normal seated eye level during setup.

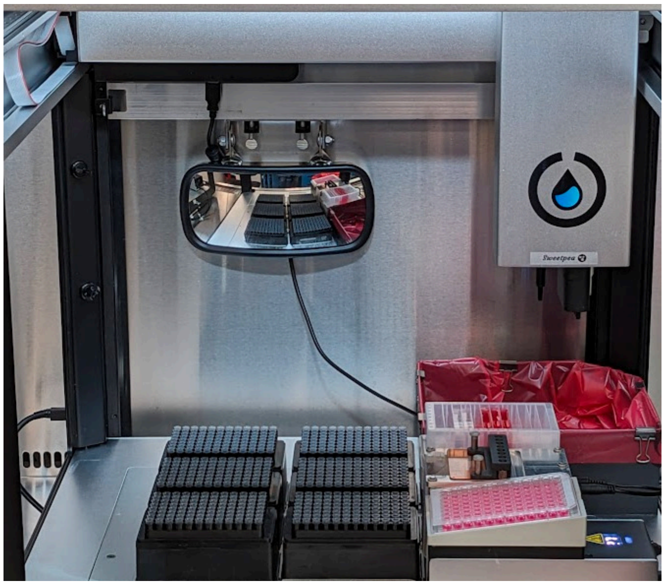

Other tools and facilities

It is recommended to keep a multi- and a single-channel manually operated P300 pipette that fit universal tips (allowing use with the Opentrons tips) with the system.

In addition, particularly if the system is installed in a BSC, it is very helpful to have a suction line and waste trap available for emptying the CCI waste cup and reagent reservoir in-place.

For clearing liquid from the CCI flow cell (see *CCI Normalization Sample SOP*), we currently use a pneumatic blow gun (McMaster-Carr 5186K81) with a non-filter 300uL pipette tip attached using tape. The blow gun is supplied by house compressed air via a regulator and particulate filter; pressure is set to ~0.2 bar. This step could also be performed with a manual pipette with potentially lower aerosol generation risk but we have found the compressed air method to yield the most consistent results in terms of the flow cell being properly primed by the robot.

OT-2 setup and modifications

Pipette modules

Pipette modules should be installed as follows, following the Opentrons documentation:

- Left mount: P300 single-channel Gen2
- Right mount: P300 8-channel Gen2

Side / top panels

Assuming the system is to be used in a biosafety cabinet, the clear side and top panels should be removed from the robot to allow for free air flow.

Tip waste bin removal

To accommodate the custom tip waste bin, the built-in bin assembly must be removed from the OT-2. To do this, first take the black plastic insert and detach the bin frame by removing four screws in the locations indicated below:

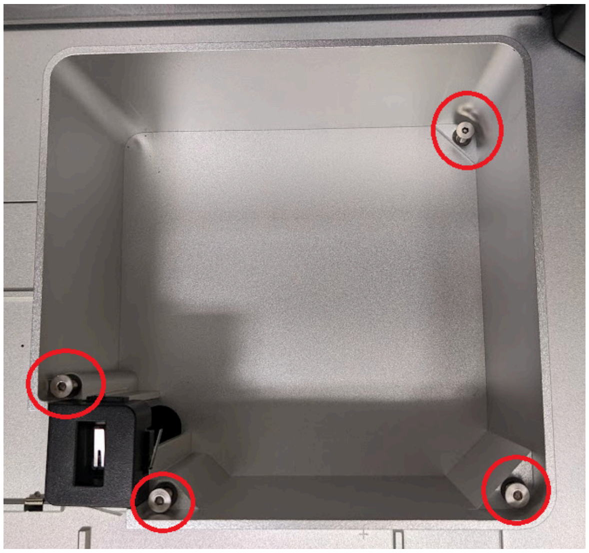

With the bin frame loose, lift it up and disconnect the electrical connector before fully removing it from the deck. Feed the end of the cable down through the passthrough hole and tape it to the underside of the deck to keep it out of the way.

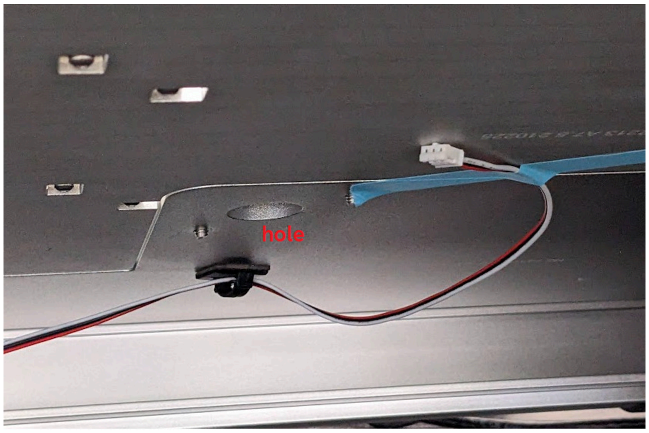

Ethernet connection

Operation of the CCI relies on a consistent network connection between the OT-2 and the PC running the CCI software. Ideally the PC and robot(s) in use should share an ethernet network and each have fixed IP addresses.

The version of the robot currently sold (sometimes called OT-2R) has an ethernet connection available and can be used as-is. Early versions of the OT-2 provided a USB port as the primary hardwired connection for controlling the robot. Internally this is wired to a USB ethernet adapter which in turn is connected to the Raspberry Pi that runs the robot. This can be removed to use a direct ethernet connection instead.

Remove the left hand frame panel from the OT-2 to expose the compartment with the ethernet and power connections in it. Unplug the USB-ethernet adapter from the PC board and the internal ethernet patch cable. Thread a 1.5ft male-female ethernet extension cable (e.g. Cable Matters 160024-BLK-1.5) through the bottom of the robot frame into the compartment and connect it to the internal patch cable with a female-female coupler (e.g. Monoprice 107297).

Ready-to-use ethernet jack on OT-2R:

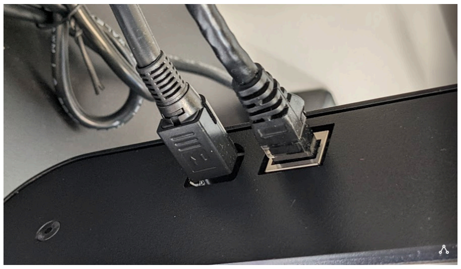

Old style OT-2 with USB ethernet adapter:

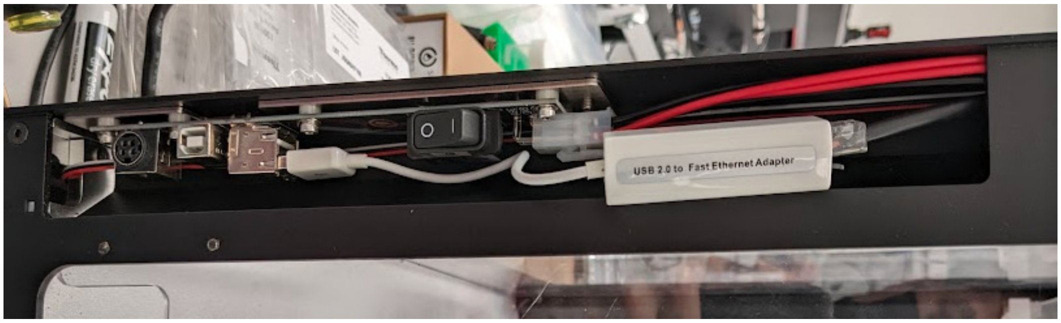

USB ethernet adapter removed and replaced with coupler and extension:

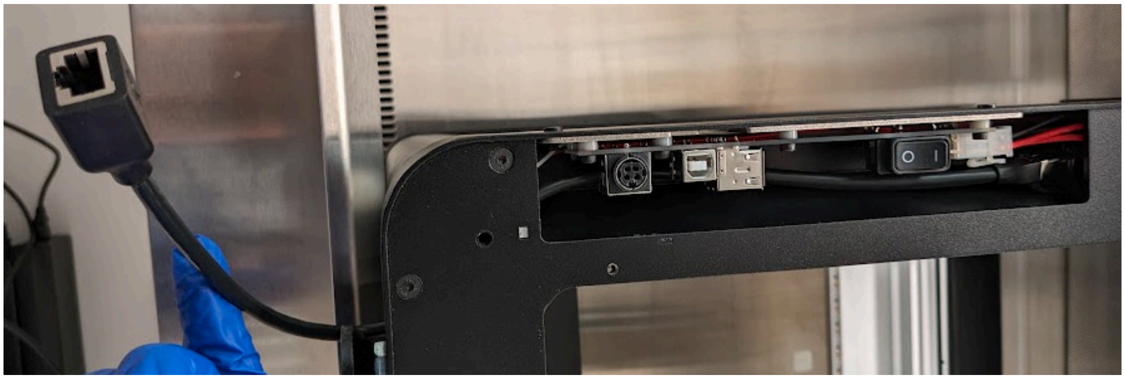

Software environment

The software environment on the robot is based on Opentrons OT-2 4.7.0 software image. The system has not yet been tested and validated with later Opentrons software versions. A new OT-2 must be downgraded by following the [instructions on the Opentrons site](#).

The Opentrons app and matching robot software image version 4.7.0 can be found here: https://github.com/Opentrons/opentrons/releases/tag/v4.7.0

The ACCS protocol framework repository includes `ot2logbot` as well as instructions for obtaining and installing the appropriate Opentrons software version on the OT-2 and setting it up for ACCS operations:
https://github.com/czbiohub-sf/accs-protocol-framework-pub

After installing the 4.7.0 software image, the only further change that is strictly required is to install the `requests` Python package, which is used to communicate with the CCI server. Optional components such as the ACCS Slack alert service (`ot2logbot`) can be installed by following the accompanying READMEs.

The above can be achieved using the file upload facility and terminal emulator available in the onboard Jupyter environment, accessible via a link in the Opentrons app. For more convenient access, particularly for transferring files, it is helpful to set up SSH access to the OT-2 by following the instructions supplied by Opentrons.

Hardware installation

Deck layout

The photo below shows a typical layout of equipment installed on the OT-2 deck for the cci_normalization protocol.

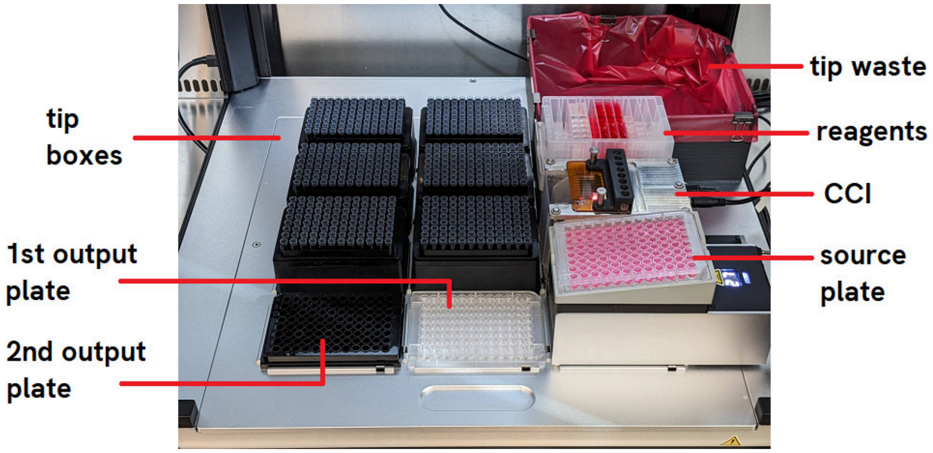

See the *CCI Normalization Sample SOP* for more details on installing items on the deck for each run.

The CCI, reservoir riser and Temperature Module are normally always left on the deck unless running protocols that require other items to be installed in their corresponding deck slots.

Installation in a biosafety cabinet

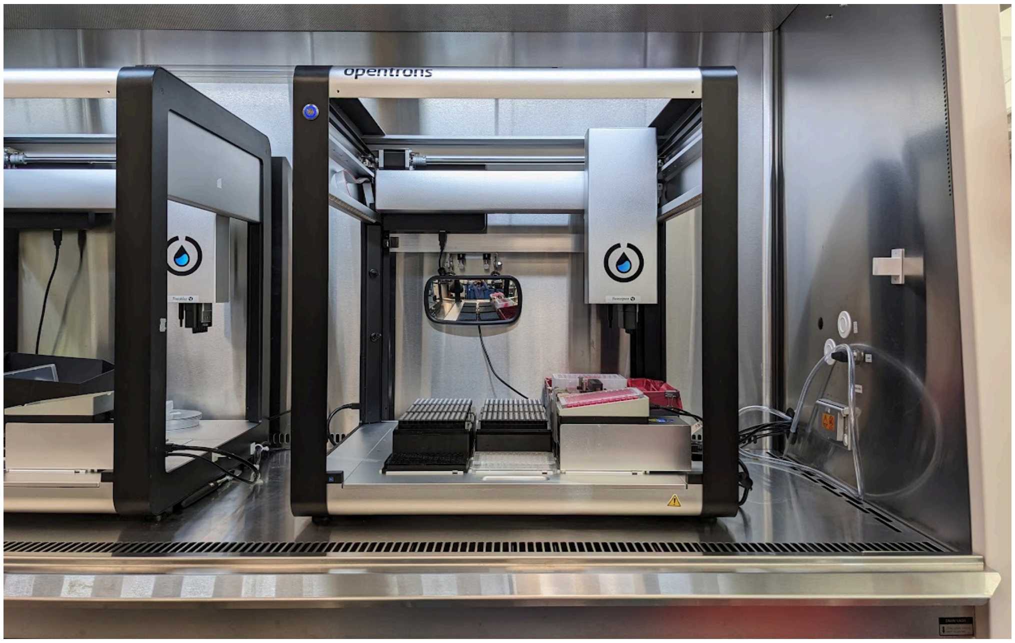

A single ACCS setup can comfortably occupy a typical 4-foot biosafety cabinet with enough workspace left on the side for manual prep tasks such as filling the reagent reservoir. Two ACCS setups can fit side-by-side in a 6-foot cabinet, but using the same cabinet for prep work becomes less practical, especially when both robots are in use. Additionally, the work surface must be rated (or appropriately reinforced) to support the weight of the two robots and all other equipment.

Note that the OT-2 is heavy (48 kg), so manipulating it in and out of the cabinet should be done using at least two people.

Also note that reaching the back of the OT-2 deck in a BSC with the sash at normal operating height may be challenging depending on the length of the operator's arms. In this case the operator may consider temporarily opening the sash fully to pre-install the trash bin and (lidded) rear row tip boxes.

Power and electronic connections

The following wiring must be routed into the BSC, per setup:

- OT-2 power cable
- Temperature Module power cable
- OT-2 ethernet cable

- CCI camera ethernet cable
- CCI stage power cable
- CCI stage serial cable

Wiring and support equipment for the CCI is covered in the *ACCS Cell Counting Imager Technical Manual*.

To save space and reduce airflow obstructions in the biosafety cabinet, we locate the power supplies for the OT-2 and Temperature Module outside the cabinet. Both devices use identical 36V power supplies whose DC output leads terminate in a Kycon KPPX-4P connector. We use off-the-shelf 4ft extension cable assemblies (GlobTek KPPX4124642M0KPJX4(R)) to add the necessary length to the output leads to route them into the BSC.

PC software setup

The first software to install is the Opentrons app, specifically version 4.7.0, which is available on GitHub:
https://github.com/Opentrons/opentrons/releases/tag/v4.7.0

The ACCS software suite includes the ACCS protocol framework itself, the user-facing UI for generating protocol scripts, and tools for protocol development. The latest version of the software and instructions for setting up the system can be obtained from the Github repository: https://github.com/czbiohub-sf/accs-protocol-framework-pub . This repository also includes the optional Slack alert bot that can be installed on the OT-2 (`ot2logbot`).

Refer to the *CCI Technical Manual* for the setup steps for the CCI server, camera drivers, etc.

Equipment list

Core off-the-shelf equipment

- Opentrons OT-2 pipetting robot, 999-00111
- Opentrons 8-channel P300 Gen2 pipette, 999-00006
- Opentrons 1-channel P300 Gen2 pipette, 999-00003
- Opentrons Temperature Module Gen2, 991-00350-0
- Operator PC with at least 1 gigabit ethernet port

Tools

- Universal fit P200 or P300 single-channel manual pipette
- Universal fit P200 or P300 8-channel manual pipette
- Telescopic inspection mirror, e.g. McMaster-Carr 1017T25
- Pneumatic blow gun -- see Other tools and facilities

Custom components

See corresponding sections under Hardware Components for detailed information

- 3-0390 Tip waste bin
- 3-1482 Reservoir riser
- 3-0271 Tilted heat block adapter
- 3-0938 Tilted plate calibration tool
- Cell Counting Imager and accompanying components:
  see ACCS Cell Counting Imager Technical Manual

Other

- 2x DC power extension lead for OT-2 and Temperature Module, GlobTek KPPX4124642M0KPJX4(R)

Consumables

Lab plastics

- Pipette tips: OT-2 200µL Filter Tips, Opentrons 999-00081
- Culture plate: BioLite 96-well plastic microwell plate, Thermo-Fisher 130188

Cleaning and disposal supplies

- Tip trash liner: 19" x 23" biohazard bag (modified as described in the Hardware Components section), Heathrow Scientific HS10322
- CCI flow cell external cleaning agent: Blue Ribbon Products Plexi-Clean
- Wipes for CCI flow cell external cleaning: 4x4" polyester cleanroom wipes, Texwipe Absorbond TX404

Reagents

Refer to the *CCI Normalization Sample SOP* for more context for the following.

- Buffer for cell wash and CCI flow cell flush: Dulbecco's Phosphate-Buffered Saline, Gibco 14190-144
- Dissociation agent: 0.25% Trypsin-EDTA, Gibco 25200-056
- Culture media (example as used for routine culture of HEK293):
    - DMEM, high glucose with GlutaMAX, Gibco 10566-016
    - + 10% v/v fetal bovine serum
    - + 25 mM HEPES, Gibco 15630-080
    - + 100 U/mL penicillin-streptomycin, Gibco 15140-122
- CCI flow cell deep clean solution: 10% v/v in DI water of Contrad 70, Fisher Scientific 04-355
- Deionized water for final CCI flow cell rinse

Note in particular the addition of HEPES to the media as a proactive measure to stabilize culture pH as plates may spend multiple hours outside of the incubator.

Supplement C.

CCS Cell Counting Imager Technical Manual

- Intro, scope and first steps
- Bill of Materials
  - Custom machined / printed parts
  - Off-the-shelf hardware
- CCI instrument machined / printed parts
  - Chassis parts (3-1239, 3-1240, 3-1241, 3-1242)
  - Optics platform parts (3-1243, 3-1246, 3-1247, 3-1250)
  - Motor pulley (3-1263)
  - Pipette tip guide / flow cell clamp (3-0885)
  - Darkfield illuminator parts (3-1253, 3-1254, 3-1261)
  - Focus locking sleeve and focus knob (3-1252, 3-1260)
  - Waste trough (3-1273)
- CCI instrument hardware build guide
  - Flow cell fasteners
  - Belt clamp
  - Focus locking sleeve
  - Motor controller board
  - USB serial communications cable
  - Wiring harness prep
  - Darkfield illuminator collar
    - Threaded insert
    - LED installation and wiring
  - "Middle section" and motor test
  - Linear guide mounting
  - Optics platform
  - Turret and camera tube assembly
  - Optical system
  - Drive train
  - Install focuser and illuminator
  - Top plate
- Commissioning
  - Software setup
  - Basic stage function test
  - Camera test
  - Focus adjustment
  - Top plate position adjustment
  - Software stage alignment
- CCI flow cell design and fabrication
  - Overview
  - Top (3-0352) and bottom (3-0305) plates
  - Spacer layer (3-0302)
  - Pipette tip interface (3-0303)
  - Waste nozzle strip (3-0377)
- General operating procedures
  - Using the CCI
  - Flow cell storage and working life
  - Flow cell cleaning
  - Cleaning the CCI instrument

Intro, scope and first steps

The Cell Counting Imager is an open-source imaging-based cell counter designed to facilitate automated cell culture with a pipetting robot. It is part of a project called the Automated Cell Culture Splitter (ACCS), which uses an Opentrons OT-2 to passage (harvest and transfer a portion to a new vessel with fresh media) human cells cultured in 96-well plates. This document is one of several resources being released alongside the manuscript and its purpose is to be the primary source of information pertinent to building and operating the CCI hardware.

There is no specialized experience strictly required to build the CCI, assuming the fabrication of the custom fabricated parts is outsourced. However, it should be noted that this is an experimental, unpolished design, and someone who intends to build a CCI using this information would benefit greatly from a basic foundation of general hardware prototyping and troubleshooting experience. This document does not instruct on general skills such as soldering or splicing and terminating cables. The builder should seek out appropriate resources and assistance according to their experience level in order to complete the work in accordance with relevant best practices.

It is recommended to review the main manuscript and the ACCS Integrator's Manual in addition to this document for context. Links to the preprint, written supplements and other resources such as a CAD model of the instrument can be found here:
https://github.com/czbiohub-sf/2024-accs-pub

Bill of Materials

This section enumerates all of the physical components and some specific tooling involved in constructing the CCI instrument. It assumes a ready supply of general electronics prototyping supplies such as hookup wire, heat shrink tubing, solder, etc. as well as access to basic soldering equipment and hand tools.

Custom machined / printed parts

See the following section (Custom hardware components) for further detail on each part.

Glossary: FDM = Fused Deposition Modeling (filament based 3D printer), SLA = Stereolithography (resin based 3D printer)

Part no	Description	Process	Material
Chassis			
3-1239	Frame bottom plate	CNC milling	Aluminum 7075-T651
3-1240	Frame vertical plate	CNC milling	Aluminum 6061-T651
3-1241	Frame top plate	CNC milling	Aluminum 6061-T651
3-1242	Frame shelf	CNC milling	Aluminum 6061-T651
Stage			
3-1243	Optics platform base plate	CNC milling	Aluminum 7075-T651
3-1245	Optics platform belt clamp body	SLS printing	Glass reinforced polyamide
3-1247	Mirror cube mounting bracket	CNC milling	Aluminum 6061-T651
3-1250	Stage homing switch flag	FDM printing	PLA
3-1263	Stage motor pulley	Manual drill & ream	McMaster # 1375K29
Optical system			
3-1246	Optics tube spacer ring	Manual turning	McMaster # 8978K937
3-1252	Focus locking sleeve body	SLA printing	Formlabs Tough 2000 resin
3-1260	Focus knob	SLA printing	Formlabs Tough 2000 resin
3-1253	Illuminator main body	SLA printing	Formlabs Tough 2000 resin
3-1254	Illuminator shroud, front half	SLA printing	Formlabs Tough 2000 resin
3-1261	Illuminator shroud, rear half	SLA printing	Formlabs Tough 2000 resin
Misc			
3-1273	Waste trough	SLA printing	Formlabs Clear V4 resin
Flow cell			
3-0302	Flow cell spacer layer	CO2 laser cut	(see notes)
3-0303	Flow cell pipette tip interface	SLA printing	Cast PUR
3-0305	Flow cell bottom slide	CO2 laser cut	Cell cast PMMA sheet
3-0352	Flow cell top slide	CO2 laser cut	Cell cast PMMA sheet
3-0377	Flow cell waste nozzle strip	CO2 laser etch & cut	Adhesive backed PTFE film

Off-the-shelf hardware

Qty	Vendor	Part number	Description	Drive Size
Flow cell fasteners				
2	McMaster-Carr	91125A613	SS round F-F standoff, 4-40, 1/4" OD	
2	McMaster-Carr	91830A101	SS thumbscrew, 3/8" head dia, 1/8" shldr ht, 1/4" lg	
2	McMaster	92785A507	SS cone point set screw, 4-40, 3/4" lg	0.050" hex
Chassis				
2	McMaster-Carr	1873N51	Nylon cable tie anchor, 0.13" tie wd, #4 screw hole	
2	McMaster-Carr	91075A228	SS M-F hex standoff, 4-40, 3.75" lg	1/4" ext hex
5	McMaster-Carr	91525A314	SS washer, #4, 0.344" OD	
2	McMaster-Carr	92196A107	SS socket head screw, 4-40, 5/16" lg	3/32" hex
3	McMaster-Carr	92196A109	SS socket head screw, 4-40, 7/16" lg	3/32" hex
2	McMaster-Carr	92314A110	SS hex head screw, 4-40, 1/2" lg	3/16" ext hex
3	McMaster-Carr	92314A112	SS hex head screw, 4-40, 5/8" lg	3/16" ext hex
2	McMaster-Carr	92949A106	SS button socket head screw, 4-40, 1/4" lg	1/16" hex
2	McMaster-Carr	96246A094	Heli-Coil insert, 4-40, 0.224" lg, dry film lubricated	
Stage mechanical				
1	McMaster-Carr	1679K626	MXL timing belt, 1/4" wd, 135T	
3	McMaster-Carr	3693N14	HTD idler pulley, 15mm OD, for 5mm shaft	
6	McMaster-Carr	91292A004	SS socket head screw, M2x0.4mm, 4mm lg	1.5mm hex
10	McMaster-Carr	91292A340	SS socket head screw, M2.6x0.45mm, 6mm l	2mm hex
2	McMaster-Carr	91585A093	SS dowel pin, 1mm dia, 20mm lg	
2	McMaster-Carr	92141A005	SS washer, #4, 0.312" OD	
1	McMaster-Carr	92196A022	SS socket head screw, 4-40, 9/32" lg	3/32" hex
2	McMaster-Carr	92210A107	SS countersunk socket head screw, 4-40, 5/16" lg	1/16" hex
1	McMaster-Carr	93615A111	SS low profile socket head screw, 4-40, 3/8" lg	0.050" hex
2	McMaster-Carr	93615A317	SS low profile socket head screw, 8-32, 1/4" lg	5/64" hex
1	McMaster-Carr	96246A046	Heli-Coil insert, Nitronic 60, 4-40, 0.168" lg	
3	Misumi	DBTS3-11-6-PC	Low hd shldr screw, M3, 5mm x 11mm shldr, 6mm thd	2mm hex
1	Misumi	SSE2B6-85-MC	Mini linear guide assy, 85mm lg, dual carriage	
1	Misumi	SSELB6-55-MC	Mini linear guide assy, 55mm lg, long carriage	

3	Misumi	WSSS8-5-1	Cylindrical spacer, 8mm OD, 5mm ID, 1mm ht	
1	Partsbuilt 3D	R2C2-SPRING-BELT	Belt tensioner spring	

Stage electronics

1	Pololu	3138	Tic T249 stepper motor controller	
9	TE Connectivity	1-104480-6	AMPMODU crimp socket contact for 22-26AWG	
4	TE Connectivity	1-104481-3	AMPMODU crimp socket contact for 28-32AWG	
2	TE Connectivity	104257-1	AMPMODU locking connector housing, 2 pos	
1	TE Connectivity	104257-3	AMPMODU locking connector housing, 4 pos	
1	TE Connectivity	104257-9	AMPMODU locking connector housing, 10 pos	
2	TE Connectivity	5-103908-1	Shrouded locking vertical header, 2 pos	
1	TE Connectivity	5-103908-3	Shrouded locking vertical header, 4 pos	
1	Tensility Intl	54-00063	Panel mount barrel jack, 5.5x2.1mm	
1	TE Connectivity	6-104935-0	Shrouded locking right angle header, 10 pos	
3	McMaster-Carr	91116A240	SS washer, M2, 7mm OD	
3	McMaster-Carr	91292A833	SS socket head screw, M2x0.4mm, 10mm lg	1.5mm hex
2	McMaster-Carr	92095A104	SS button socket head screw, M2x0.4mm, 10mm lg	1.3mm hex
2	McMaster-Carr	92196A082	SS socket head screw, 2-56, 7/16" lg	5/64" hex
2	McMaster-Carr	92320A453	SS unthreaded spacer, #2, 1/8" OD, 5/32" lg	
1	Omron	D2F-01L3-D	SPDT microswitch	
1	Soyo	SY20STH42-0804A	NEMA 8 stepper motor, 5.4Ω, 4.2 oz in	
1	TE Connectivity	T4070014041-001	M8 connector, 4 pos rear mount, wire leads	

Optical system

1	Edmund Optics	49-030	Longpass filter 700 nm 12.5mm	
1	Edmund Optics	67706	4x finite conjugate RMS	
1	Basler AG	acA5472-5gm	20MP 1" monochrome GigE camera	
4	McMaster-Carr	92196A106	SS socket head screw, 4-40, 1/4" lg	3/32"
1	McMaster-Carr	99091A190	Black nylon hex head screw, 8-32, 1/4" lg	1/4" ext hex
1	Thorlabs	CCM5-G01	ThorLabs CCM5-G01 protected turning mirror	
1	Thorlabs	SM05A1	C-mount camera to SM05 male threads adapter	
1	Thorlabs	SM05L05	SM05L05 lens tube, 0.5" thread dp	
1	Thorlabs	SM05M20	SM05 Lens Tube Without External Threads, 2" Long	
2	Thorlabs	SM05RC	Slip mount for SM05 lens tube	

1	Thorlabs	SM05V05	Ø1/2" Adjustable Lens Tube, 0.31" Travel	
1	Thorlabs	SM1A1	SM1 to SM05 adapter	
1	Thorlabs	SM1A3	Internal RMS to external SM1 adapter	
1	Thorlabs	SM1L05	SM1 lens tube, 0.5" lg	

Illuminator and focus lock

8	Marktech	MTE1074N1-R	NIR T1-3/4 LED, 4mW, 740nm peak	
1	LEDynamics	4006-030	Inline current regulator module, 30mA	
1	McMaster-Carr	1173N031	Silicone O-ring, 1/16" wd, 1.88" OD	
2	McMaster-Carr	90669A074	SS Brass-Tip Set Screw, 2-56, 1/8" lg	0.035" hex
3	McMaster-Carr	92785A053	SS cone point set screw, 2-56, 3/16" lg	0.035" hex
5	McMaster-Carr	92395A311	Flanged split brass threaded insert, 2-56, 0.136" lg	

Specialty material stock or parts to be modified

1	McMaster-Carr	1375K29	10 tooth MXL pulley, for motor pulley 3-1263
1ft	McMaster-Carr	6720T12	Bend-and-stretch-rated cable stock for illuminator
1	FTDI Chip	TTL-232RG-VSW5V-WE	USB to TTL serial cable, 5V, bare wire ends
1	Tensility Intl	10-05920	M8 cable assy, 4 pos A key socket, 3 m, bare wire

Support equipment

1	Globtek	TR9KI2700LCPCIMR6B	24V 65W power supply
1	TP-Link	TL-PoE160S	30W PoE+ injector
	*	*	Computer with gigabit ethernet port
	*	*	Add'l cabling as necessary for pwr, ethernet, etc

Utility / misc.

1	ThorLabs	SM05L30	Stackable 1/2" lens tube, 3" internal thread length. Used as an alignment tool.
1	McMaster-Carr	3009A118	(or similar) 1/8" drill rod for use as press tool
1	Henkel	680522	Loctite 454 (or similar) thixotropic CA adhesive
1	Henkel	2765219	Loctite 7452 (or similar) cyanoacrylate accelerator
	*	*	General essentials and consumables not listed: Hookup wire, heat shrink tubing, etc.

CCI instrument machined / printed parts

This section briefly describes the individual machined and/or printed parts used in the construction of the CCI instrument. The CCI flow cell components are covered separately in the CCI flow cell fabrication section.

Refer to the corresponding Onshape CAD models for part geometry for manufacturing. In cases where the solid model itself does not fully describe the part requirements a 2D drawing or other supplemental files(s) will be included in the Onshape document. When uploading models to a prototype manufacturing service it is important to make sure relevant details such as threaded holes are communicated correctly, and the process differs between vendors.

Refer to the Hardware build guide section for more context regarding the function of each part and order of assembly.

Chassis parts (3-1239, 3-1240, 3-1241, 3-1242)

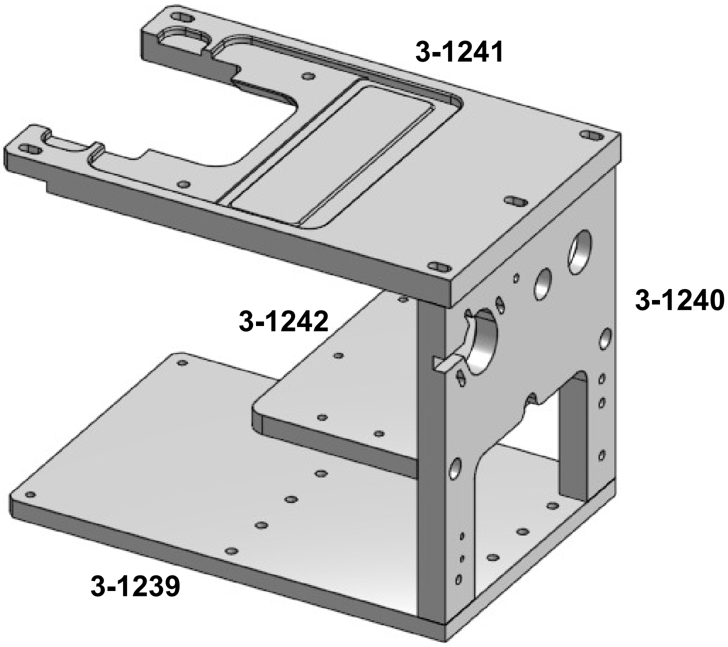

The structural frame includes four CNC machined aluminum plates:

#	Description	Material
3-1239	Bottom plate	Aluminum 7075-T651

3-1240	Vertical plate	Aluminum 6061-T651
3-1241	Top plate	Aluminum 6061-T651
3-1242	Shelf	Aluminum 6061-T651

The 7075 alloy was selected for the bottom plate on our prototypes for increased stiffness, however the corrosion resistance of 6061/6063 may be preferable due to likely exposure to spilled media, etc.

The shelf (3-1242) primarily serves as a platform for the motor control board and attachment point for the illuminator power cable and is not a critical structural component, so a printed part could be substituted to save cost.

Optics platform parts (3-1243, 3-1246, 3-1247, 3-1250)

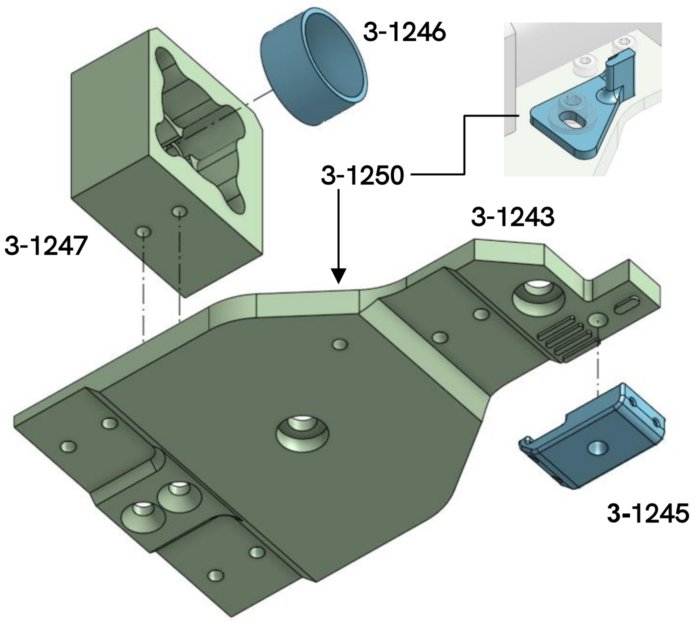

The optics platform / carriage structure consists principally of two machined aluminum parts:

#	Description	Material
3-1243	Optics platform base	Aluminum 7075-T651
3-1247	Mirror cube bracket	Aluminum 6061-T651

7075 alloy was selected for the base plate for its mechanical properties but 6061 could be substituted.

The spacer ring (3-1246) is made by cutting a 0.31" length from 5/8" OD x 0.058" wall 6061-T6 aluminum tubing (e.g. McMaster-Carr 9390N15).

The belt clamp body (3-1245) is made from SLS-printed glass-reinforced Nylon and reinforced with stainless pins (see Hardware build guide). This approach was motivated by a significant cost savings compared to ordering a CNC machined version of this small part in prototype quantities. Barring cost concerns, a solid machined part would be a simpler solution.

The homing switch flag (3-1250) was made by FDM printing in PLA plastic but the choice of material is immaterial for this part.

Motor pulley (3-1263)

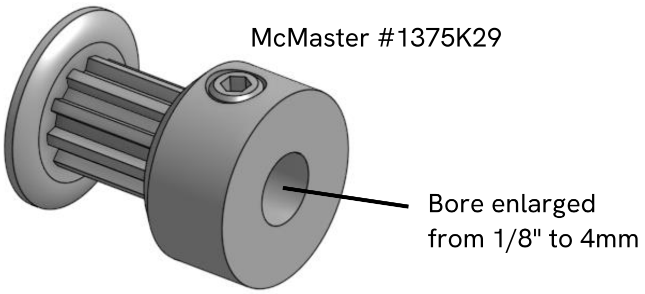

The motor pulley is made from a commercial 10-tooth, 1/8" bore aluminum MXL pulley (McMaster-Carr 1375K29), modified to fit on the 4mm diameter shaft of the stepper motor by re-drilling and reaming the bore on a lathe.

This process has proved to be troublesome and has a poor yield. The pulley is a press-fit assembly of three pieces. Enlarging the bore must be done carefully as it leaves very little material thickness in the middle piece where it mates with the hub and the end flange. In addition, any eccentricity becomes problematic for proper operation of the stage drive mechanism.

Pipette tip guide / flow cell clamp (3-0885)

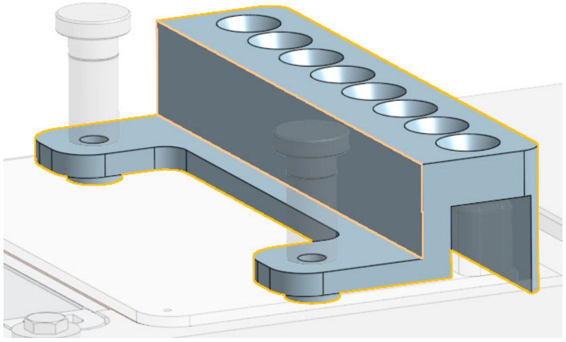

This piece is installed on top of the flow cell and held down with the thumbscrews. It was originally intended to guide the pipette tips into the relatively small holes on the pipette tip interface. The current design of the pipette tip interface itself incorporates lead-in cones to guide the tips into the openings, so now the primary purpose of the tip guide is to provide a bearing surface for the thumbscrews and provide backup protection against the pipette tip interface being stabbed by grossly misaligned pipette tips.

The pipette tip guide was made using SLA printing in Formlabs Black V4 resin. The choice of material is not critical but a dark non-reflective surface is preferable.

Darkfield illuminator parts (3-1253, 3-1254, 3-1261)

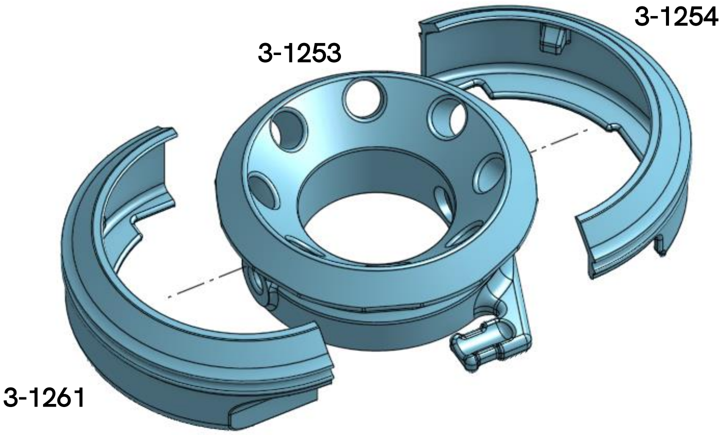

The illuminator main body (3-1253) forms the primary mechanical structure of the illuminator; the illuminator shroud front half (3-1254) and rear half (3-1261) conceal the LED wiring and provide a small amount of shielding from incidental liquid exposure.

All three parts are made using SLA printing in Formlabs Tough 2000 resin.

Focus locking sleeve and focus knob (3-1252, 3-1260)

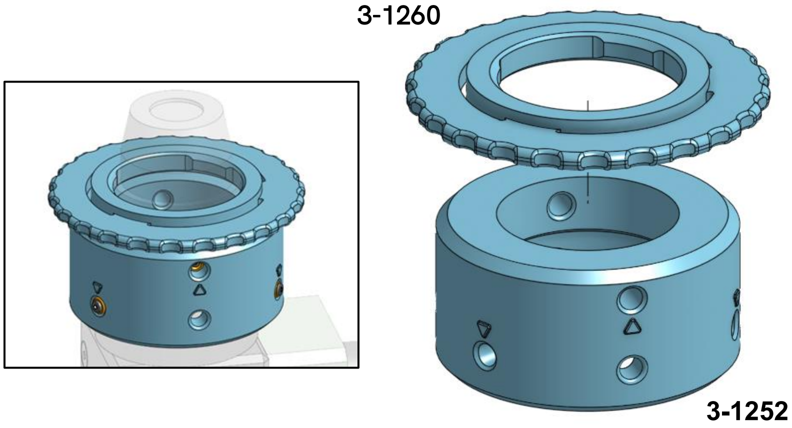

The focus locking sleeve body (3-1252) is part of a simple focus adjustment system wherein the height of the objective can be adjusted by screwing an adaptor in and out of a threaded tube and locked by jamming a set screw against the side of the objective. The part is finished by installing threaded inserts which hold 3 cone-tipped (for securing the sleeve in place on a tube) and one brass-tipped (for jamming against the objective) set screws. An access hole opposite each insert location acts as a drill guide for cleaning up the printed bore, then a press tool (a short length of 1/8" drill rod) is inserted through the access hole to press the insert into place.

The focus knob (3-1260) simply press-fits onto the outside of the objective and provides a way to rotate it with one hand while the other hand keeps the illuminator from spinning (there is no clearance for fingers to reach the objective itself in this situation).

As with the darkfield illuminator parts, both of these parts are made using SLA printing in Formlabs Tough 2000 resin. Using a material with different properties may require slight design adjustments. As discussed in the Hardware build notes section, the focus locking sleeve may require some additional post-machining.

Waste trough (3-1273)

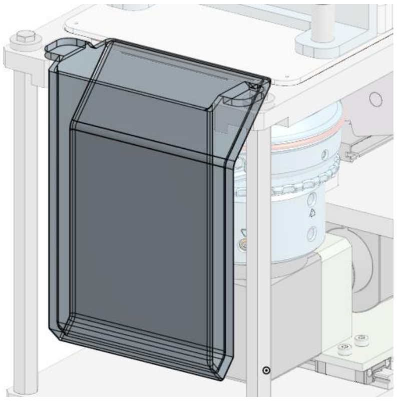

The waste trough hangs from the top plate of the CCI frame underneath the flow cell and collects the liquid that flows out of the drain ports.

The waste trough is made by SLA printing in Formlabs Clear Resin V4. It is important to use a non-opaque material so that the liquid level is readily visible, reducing the risk of overflow due to failure to empty waste from a previous run.

The waste trough could be considered a semi-disposable item, although in practice the material appears to hold up well to long term use and repeated disinfection with bleach.

A modified design with a drain port plumbed to an external waste trap could hypothetically remove the need to manually empty the trough between runs.

CCI instrument hardware build guide

The order of operations presented here is somewhat arbitrary.

Flow cell fasteners

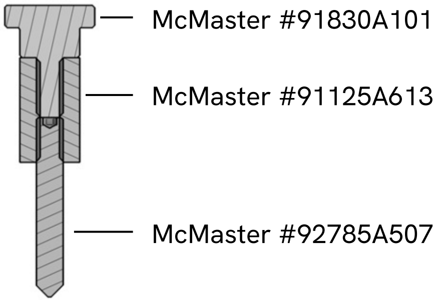

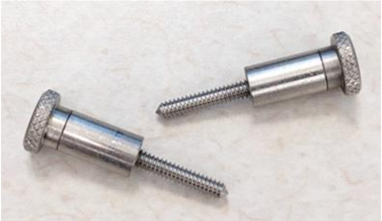

Assemble two flow cell fasteners by firmly threading together the three component parts and securing with permanent threadlocker or epoxy.

Belt clamp

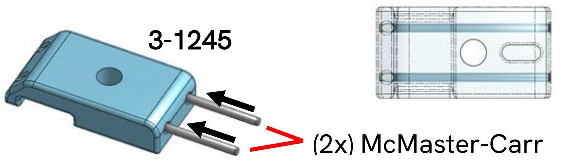

The belt clamp is completed by simply inserting two 1mm x 20mm stainless steel pins (McMaster-Carr 91585A093) into the 3D-printed belt clamp body (3-1245) using a small arbor press. If necessary, a bench vise or other tool could potentially be used instead, or the holes could be drilled for a clearance fit and the pins glued in place.

Focus locking sleeve

Completing the focus locking sleeve (3-1252) involves installing a total of four threaded inserts. Essentially the installation process is the same as for the illuminator collar but without the need to brace the part to keep it upright.

After setting the four inserts, install three cone point set screws (McMaster-Carr 92785A053) in the "lower" holes (actually on top in the pictures below) and a brass-tip setscrew (90669A074) in the "upper" hole.

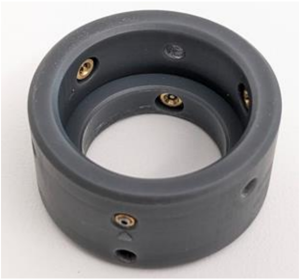

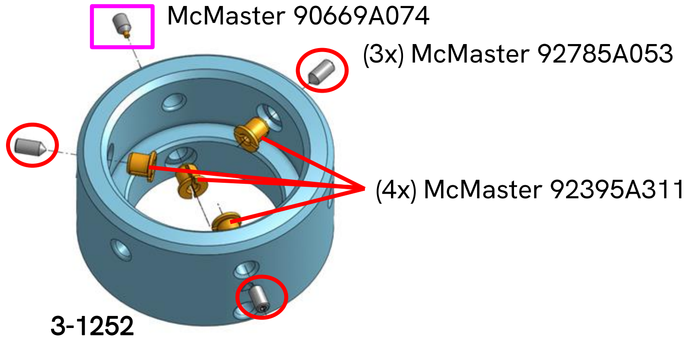

Motor controller board

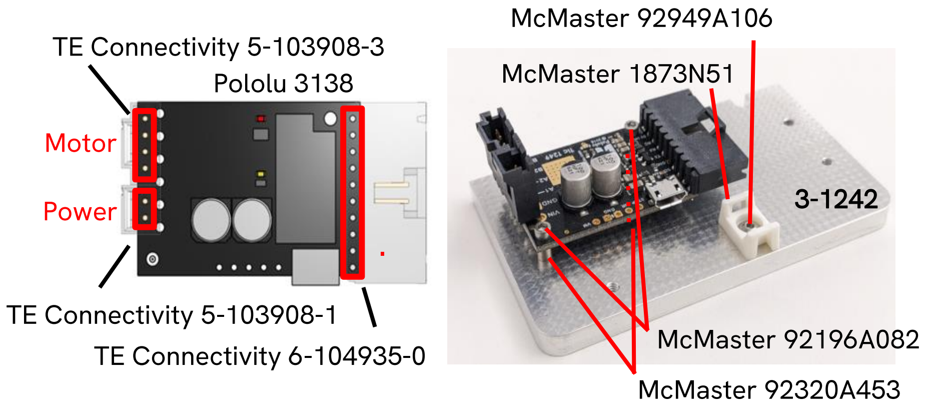

Solder three connectors onto the Tic board as indicated, then mount the board on the shelf plate 3-1242 using the specified screws and spacers.

Also mount a cable tie anchor at the indicated location.

USB serial communications cable

This cable connects the host PC to the serial interface for the motor controller on the CCI instrument. It is made by splicing an M8 socket pigtail cable (Tensility 10-05920) to an adapter cable with a TTL-level USB serial port interface in it (FTDI TTL-232RG-VSW5V-WE).

This combination gives a maximum reach of up to 15 ft if using the full length of both half cables. Cut down the length of the M8 pigtail before assembly to produce a length that is not excessive for your installation.

Only three connections are used -- the two serial data lines and signal ground. Unused wire ends on the FTDI cable side should be dealt with appropriately to ensure that they don't contact each other.

Wire color,<br>FTDI cable end	Function	Wire color,<br>M8 cable end
~~Red~~	~~+5V~~	->(don't connect)
**Black**	GND	**Black**
**Yellow**	RxD (CCI->PC)	**White**
**Orange**	TxD (PC->CCI)	**Brown**

~~Green~~	~~RTS~~	->(don't connect)
~~Brown~~	~~CTS~~	->(don't connect)
(don't connect)<-	n/a	~~Blue~~

Wiring harness prep

The **I/O harness** connects the homing switch and serial port to the 10-pin header on the Tic board. Start by wiring the N.O. side of the homing switch to positions 6~7 of the 10-position connector with at least about 7cm of movable wire length, as illustrated below.

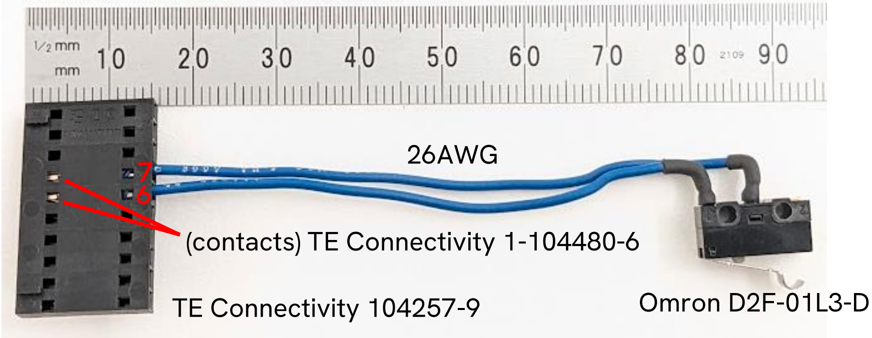

Next add the serial communications lines from the M8 bulkhead connector (TE Connectivity T4070014041-001): cut off the blue wire from the M8 connector close to the base, trim the remaining leads to about 12cm long and terminate them in the 10-position connector per the table below to complete the I/O harness.

M8 plug position no.	Wire color	Function	Header position no.
4	**Black**	Signal ground	**1**
1	**Brown**	Serial data PC→Tic	**4**
2	**White**	Serial data Tic→PC	**5**
n/a		Switch COM*	6
n/a		Switch NO*	7
			* interchangeable

Next wire the **power harness** with the barrel jack and two two-pin connectors in parallel as shown below.

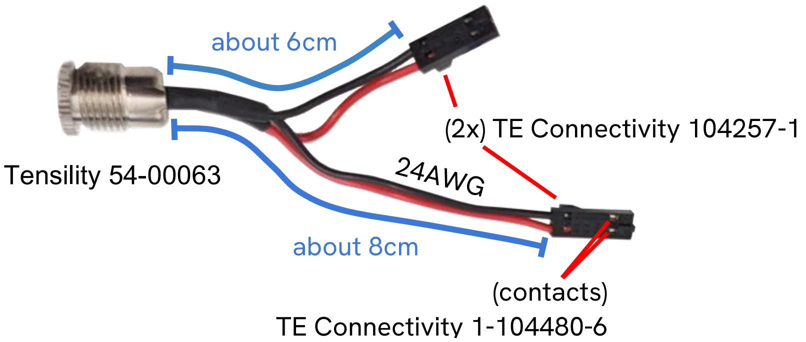

Barrel jack terminal	Function	Header position no.
Outer	Ground	1
Inner	+24V	2

Note that although the barrel jack is a front-mount design, the 2-pin connectors will fit through the mounting hole for the jack, so the harness does not have to be assembled in-place.

Finally, terminate the **motor cable** included with the stepper motor with a 4-pin connector as shown:

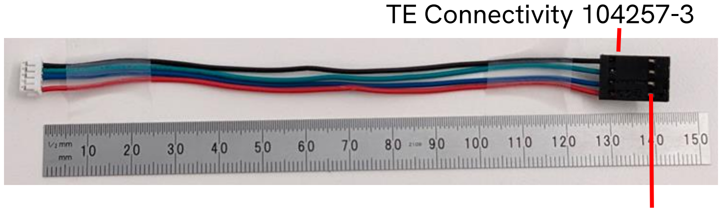

Wire color	black	green	blue	red
Header pos. no.	1	2	3	4

Note that the motor leads are 28AWG which requires different contacts than the previous 3 connectors.

Darkfield illuminator collar

Note: Before proceeding to build the illuminator, check the fit of the LED holes by inserting an LED into each one and verifying that the LED passes freely into the hole and that the flange of the LED bottoms out squarely on the flat land surrounding the hole. This should be done first in case any rework of the part is needed.

Threaded insert

The illuminator collar slips over the end of the objective and is secured in place by bearing down on it with a soft-tipped setscrew. The setscrew is installed in a flanged threaded insert which is installed from the inside of the bore. The access hole opposite the threaded insert provides access for a press tool but the protruding rim of the part presents a challenge when attempting to apply force in a controlled manner. The design includes a small flat boss around the through hole which can be used to brace the part against a suitable object such as a square wooden bar as illustrated below.

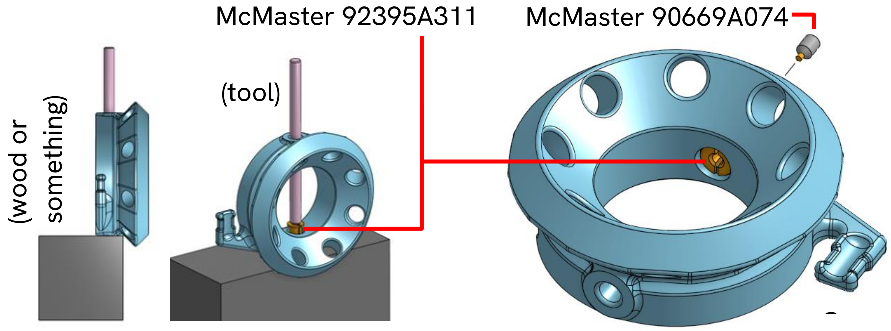

Before attempting to install the insert, run a 1/8" drill through the two opposing holes in the base of the part as a "clean-up pass" in case they are undersized or distorted.

With the part properly supported, the insert should be able to be pressed in easily using a piece of 1/8" drill rod held in a drill press or whatever method is readily available. After installing the insert, drive in a sacrificial #2-56 cap screw (from the outside hole) to expand it. Make sure a cap screw can be threaded all the way through the insert without binding up before attempting to drive in a setscrew with a hair-thin hex key. The inserts are easily damaged by mis-installation, so it is suggested to order extra stock to avoid having to try to work with a compromised insert.

After a successful function test, install the brass-tipped setscrew (90669a074) in the insert from the outside, so that the tip faces the center bore.

LED installation and wiring

Electrically, the illuminator consists of a loop of 8 LEDs broken in the middle by an inline current regulator and supplied with 24V DC, as illustrated below.

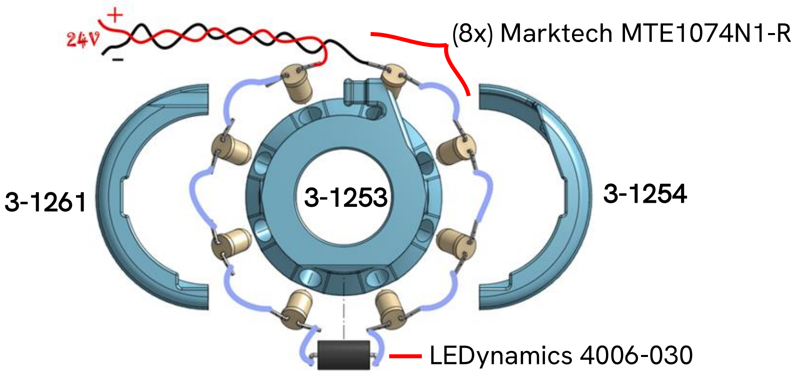

Physically, the LEDs are inserted into angled holes and held in place with glue, then the leads are bent and soldered together. Power is supplied from the instrument's power input harness via a flexible cable that moves with the stage.

We have found that the installation process is easiest when the LEDs have a free fit or a very light interference fit in the holes. If the fit is slightly too tight, the part may be adjusted by running a #8 or #7 drill through all the holes. We use a gel-type cyanoacrylate glue (Loctite 454 or similar), so a slightly loose fit is tolerable and less risky than a too-tight fit.

For each LED, apply a small blob of adhesive on top of the rim, insert the LED into the hole, and rotate it so that the flat side of the rim faces the counterclockwise direction (when viewed from above). After all 8 LEDs are placed, use tweezers or another pointed tool to poke the base of each LED and make sure it is fully seated. Once satisfactory placement of all LEDs is confirmed, apply a spray of accelerator (e.g. Loctite 7452). Additional glue can then be added on for security if needed.

Bend the leads of the current regulator at a 90 degree angle close to the body and then glue it into the curved recess in the orientation shown below, making sure the end marked "+" is facing the *clockwise* direction (opposite to the flats on the LEDs).

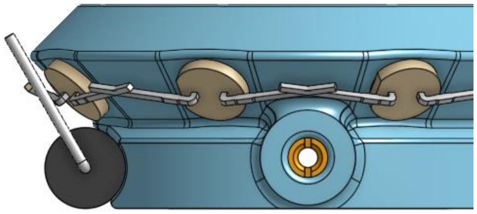

Manipulate the leads of the current regulator and its neighbor LEDs into contact and trim away as much excess material as possible. Repeat this down the chain but stop at the last pair of neighbor leads, as this is where the power cable will be connected. Test-fit the shroud halves (3-1261, 3-1254) periodically in the process in order to solve clearance issues as they arise.

Make the power cable for the illuminator by cutting a 180mm length of flex cable (McMaster 6720T12), removing about 20mm of outer jacket at each end, and terminating one end with a 2-position header (TE Connectivity 5-103908-1), following the polarity convention of the power harness made earlier (i.e. positive on pin 2). Finally, solder all of the connections around the loop and connect the power cable (red wire to the curved side of the first LED, green wire to the flat side of the last LED). Use a cable tie to secure the outer jacket of the cable to the arm for strain relief as shown.

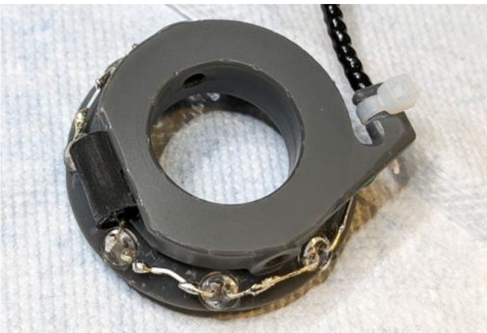

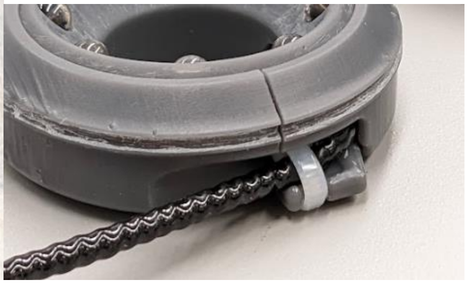

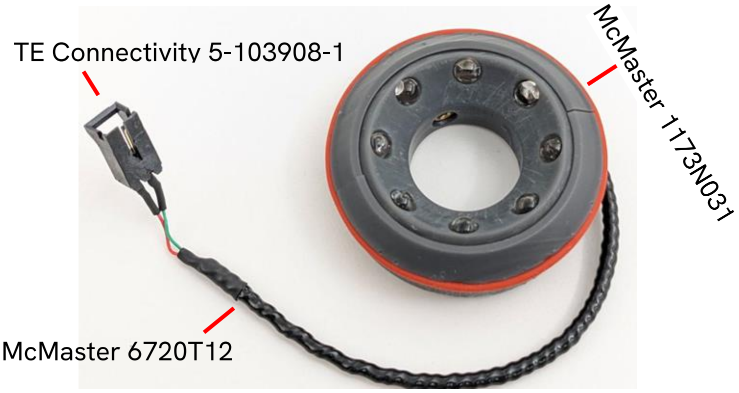

After fitting the shroud halves, the silicone O-ring (McMaster 1173N031) can be hooped around the shell halves to hold them together. Test with power to verify that the LEDs light up (the output will be visible to human vision as a dim red glow), then set the completed illuminator aside.

"Middle section" and motor test

First install three idler assemblies on the vertical plate 3-1240 as illustrated. Note that there are four holes where the idlers can be mounted; the correct three locations are indicated in the diagram below.

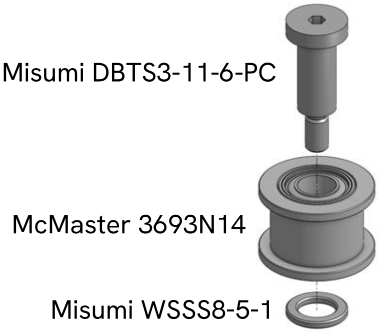

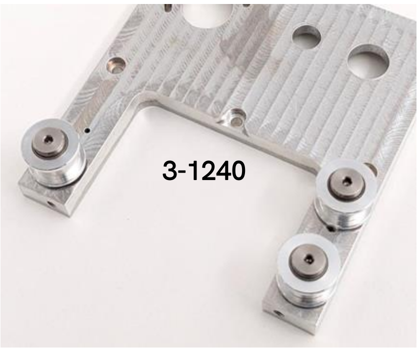

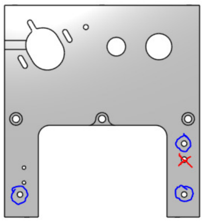

Next install the serial connector, the power connector and the homing switch on the vertical plate as shown:

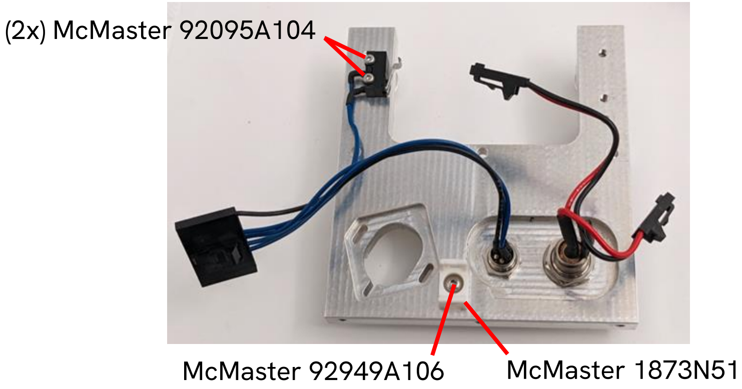

Attach the shelf plate to the vertical plate with 3 screws as indicated, taking care to route the switch wires through the notch in the shelf plate. The I/O and and motor power connectors can then be plugged in.

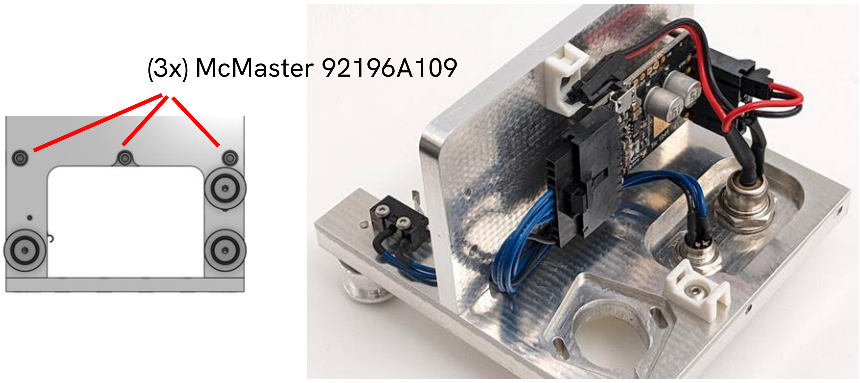

Attach the motor pulley to the motor shaft loosely (only screw in the set screw enough to key the pulley to the flat on the shaft, then back off). Install the motor on the vertical plate as shown, with the screws installed loosely so that it can move along the slots. Plug in the motor cable and secure it to the upper cable tie anchor.

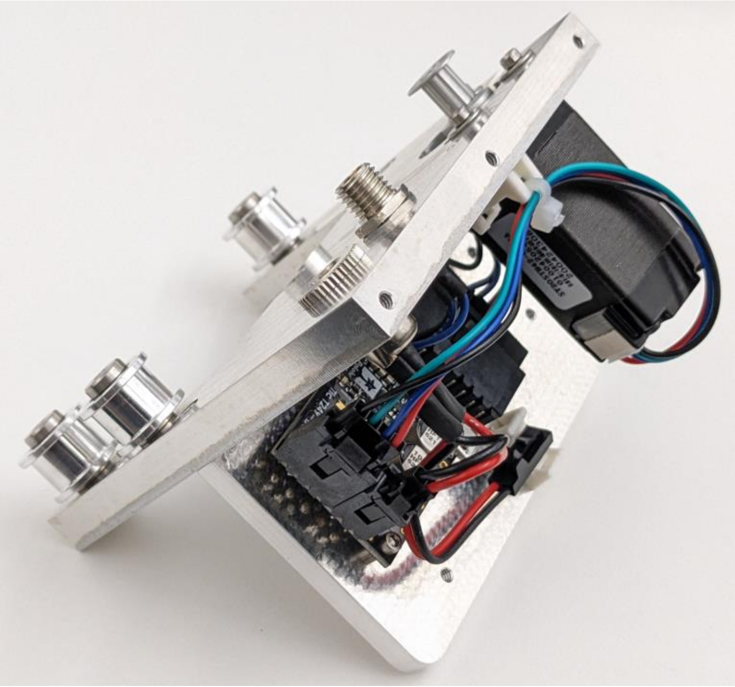

It is highly recommended to do initial setup and testing of the Tic board at this point. Find and install the appropriate software download for your OS from the manufacturer's website: https://www.pololu.com/docs/0J71/all

Connect the Tic board to a PC via its MicroUSB connector and plug a 24V power supply (e.g. Globtek TR9KI2700LCPCIMR6B) into the power connector on the bulkhead. Run the Tic Control Center software. It should immediately connect to the board and start displaying status information:

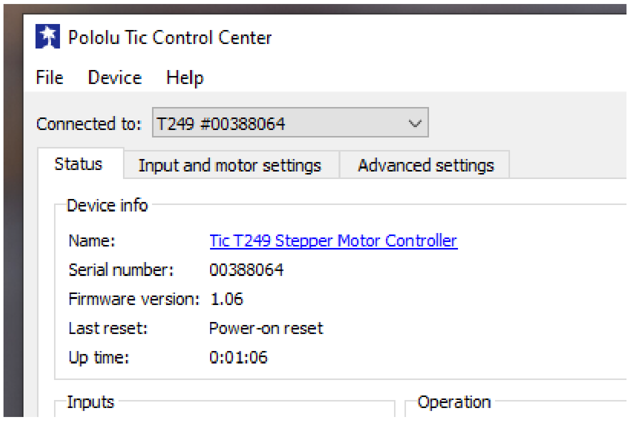

To initialize the Tic board with the correct configuration for the CCI, select "File" -> "Open settings file...", open the tic_settings_cci2.txt file obtained from the CCI GitHub repository, then use the "Apply Settings" button to send the new settings to the board.

Press the limit switch manually and you should see an indicator appear:

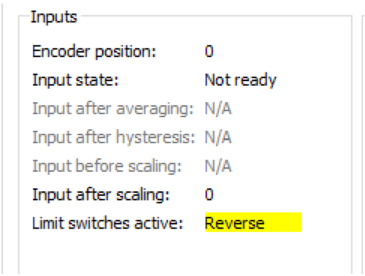

You can simulate homing the stage using "File" -> "Go home reverse". The motor should start slowly turning. Push the homing switch and it should reverse direction, then stop when you let go of the switch. Finally, you should be able to use the "Set position" and "Set velocity" controls at the bottom of the window to manually make the motor move.

Linear guide mounting

> **Warning:** Without endstops in place it is easy to inadvertently let the linear guide carriages run themselves off the rails when handing the bottom plate assembly, especially once the weight of the optics platform is attached. The carriages coming off the rails is not necessarily a catastrophic event, but the optical system meeting the floor could be. Until the "Middle Section" is attached to the bottom plate, consider using tape to avoid unexpected movement.

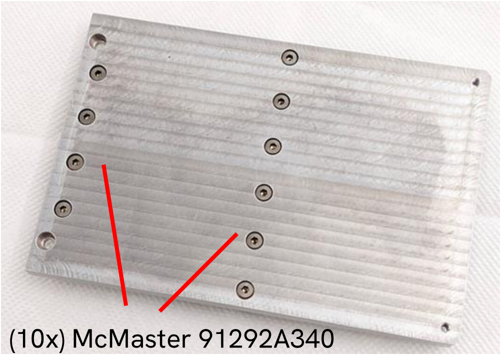

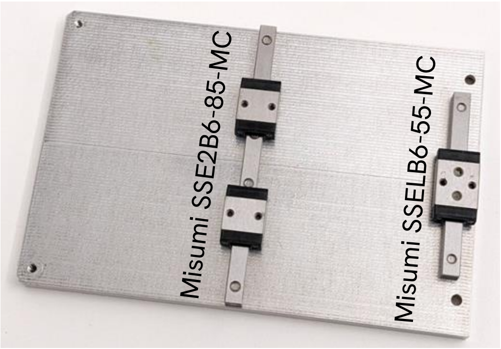

Line each linear guide rail up with its corresponding holes in the bottom plate and install all of the screws as indicated but only take up the slack, do not tighten. Looking at the bottom of the bottom plate, manually manipulate the longer rail so that all of the screw heads for that rail appear centered in the counterbores, then gradually tighten them in multiple passes from the center outward.

The shorter rail should be left slightly loose for now; it will be aligned and fastened in a later step.

Optics platform

Install the Heli-Coil insert (McMaster-Carr 96246A094) at the indicated location on the optics platform base plate (3-1243) if this operation was not ordered as part of the manufacturing of the part.

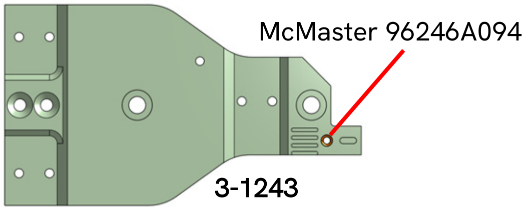

Attach the homing flag and tube clamps as shown below, but *do not fully tighten the tube clamps down to the plate yet* -- they should be able to rotate slightly but should not wiggle freely.

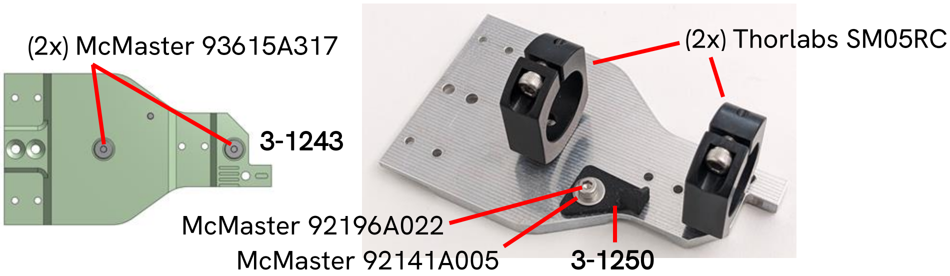

Slip a Thorlabs SM05L30 tube through the clamps to align them and tighten the clamps until they both grip the tube, then fully tighten the mounting screws for the clamps. Open the clamps and remove the tube.

Turret and camera tube assembly

Install the RMS-to-SM1 adapter (Thorlabs SM1A3) on the objective (Edmund Optics 67706). Tighten firmly and consider mounting the adapter permanently using a threadlocker.

Place the 700nm longpass filter (Edmund Optics 49-030) in the Thorlabs SM05L05 lens tube and secure it using the locking ring included with the tube.

Install the black nylon screw (McMaster 99091A190) in the open mounting hole on the housing of the turning mirror (Thorlabs CCM5-G01).

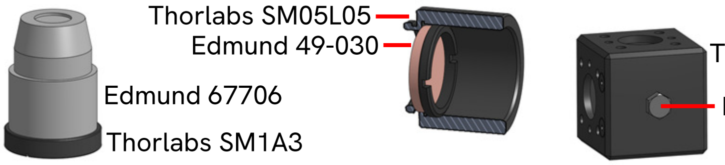

Facing the side of the mirror cube you just installed the screw in, with the openings facing up and left, screw the Thorlabs SM05-to-SM1 adapter (SM1A1) into the top hole followed by the 0.5" SM1 tube (SM1L05). Make these connections as tight as reasonably possible.

Install the mirror cube bracket (3-1247) on the side of the mirror cube with 4 screws as shown; *leave the screws loosened by 1/4 turn for now*. *Make sure the two screw holes on the outside of the bracket are facing down* (opposite from the direction the objective is pointing).

Screw the SM05L05 with the filter in it onto the end of the SM05 "adjustable" lens tube (SM05V05). Slip the spacer ring (3-1246) over the external threads and then screw the tube into the mirror block. The tube should bottom out on the spacer ring, such that the mirror cube bracket still has a small amount of translational freedom.

Thread the objective in its adapter partway down into the top tube. Do not bottom it out. The SM1 adapter and the tube are used as a crude focus adjustment mechanism.

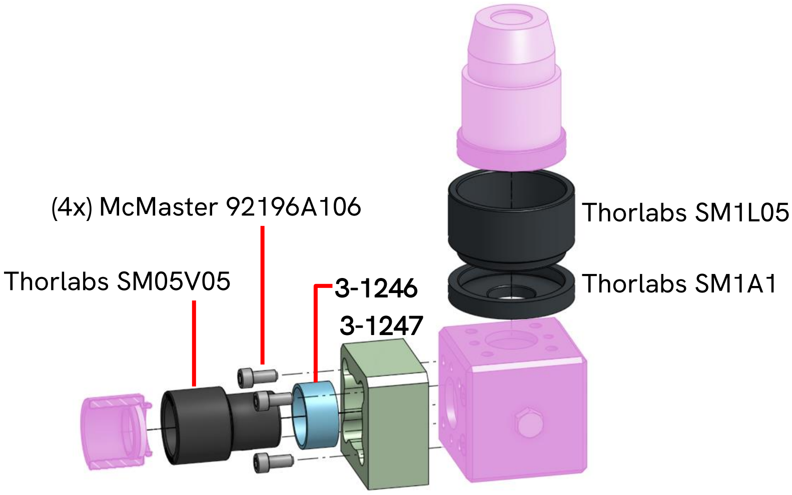

Install the C-mount adapter (Thorlabs SM05A1) tightly into the 2" SM05 tube (SM05M20). Install the adapter onto the camera (Basler acA5472-5gm).

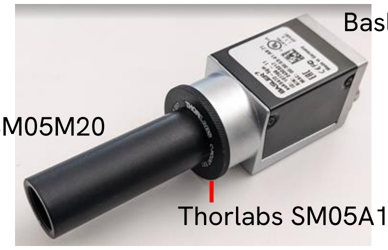

Optical system

Temporarily install the long SM05 tube (Thorlabs SM05L30) onto the open leg of the turret assembly to use for alignment. Insert the tube through the clamps. Tighten the clamps just until the tube has absolutely no wiggle but can still move axially without needing excessive force. Line up the mirror cube bracket mounting holes with the corresponding countersunk holes on the base plate. Install and tighten two screws to attach the bracket to the base plate as indicated below.

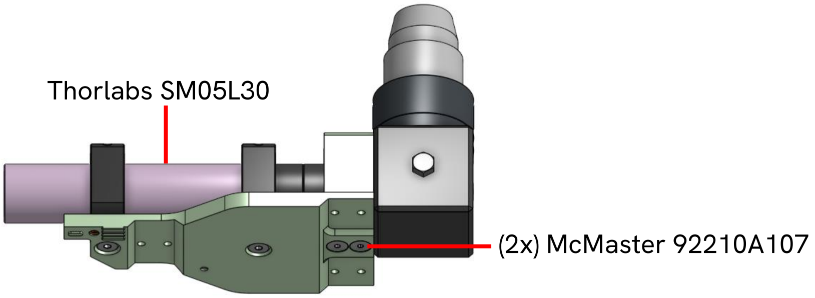

Viewing the assembly from the side opposite the long tube as shown below, make sure the turret is pointing straight up (perpendicular to the base plate) and adjust the angle if necessary. Ensure the opposite side of the cube is pushed all the way up against the cube bracket.

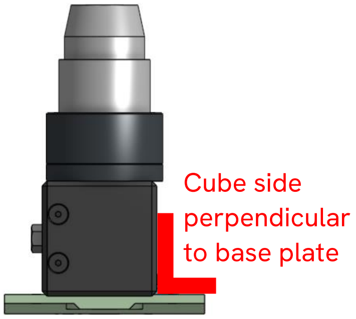

Tighten the 4 screws inside the mirror cube bracket using the short leg of a 3/32" hex key. Loosen both tube clamps and unscrew and remove the long tube.

Position the carriages of the linear guides to line up with the mounting holes on the optics platform baseplate and place the optics platform onto the linear guides. Install all 6 screws as indicated below, without tightening.

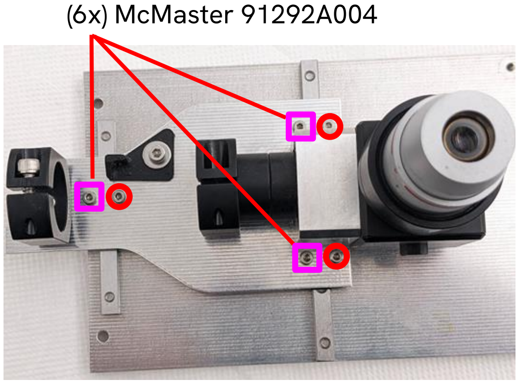

Turn the three screws designated by magenta squares until lightly snug. Move the stage back and forth from one end of travel to another. If there is excessive drag, re-loosen the three screws and try re-snugging them with less force than before while gently wiggling the stage back and forth to feel for any change in friction. Once the stage moves smoothly in this configuration, snug the screws indicated by the red circles, then fully tighten the magenta square screws followed by the rest.

Move the stage to one extreme of travel and gently tighten the outermost screw on the short rail on that side. Repeat with the stage at the other extreme. If increased drag develops at this point, try releasing and re-fastening the carriage mounting screws for the short rail. Once smooth movement is achieved, tighten all screws on the short rail in a few passes moving outward from the center.

Install the camera by sliding the attached tube through the clamps until it stops. Orient the camera as shown (silver bottom facing the same direction as the nylon screw on the mirror box) and secure it by tightening the two clamps.

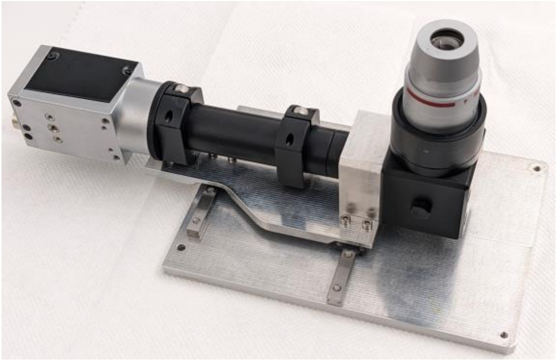

Drive train

Attach the "middle section" to the bottom plate with two screws, making sure the edges of the two parts are aligned, and then install the two long standoffs as indicated below.

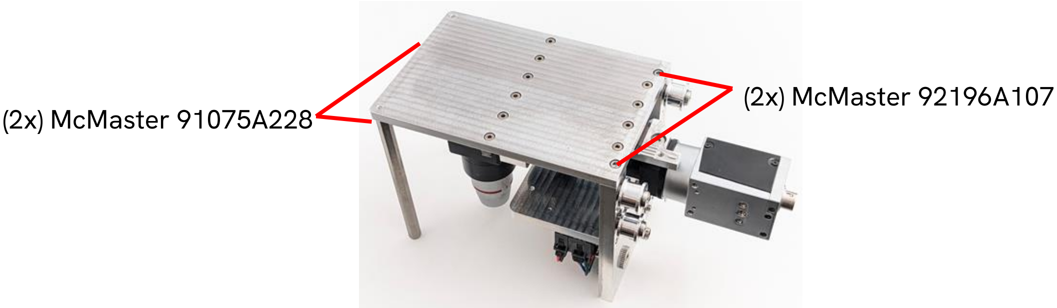

Attach the tensioner spring (Partsbuilt 3D R2C2-SPRING-BELT) to the drive belt (McMaster 1679K626) as shown. Slide the motor in its mounting slots towards the center of the vertical plate. Install the drive belt by hooking around the motor pulley and two of the idlers, then slipping it over the edge of the third roller.

Facing the outside face of the vertical plate, with the instrument upside-down, move the stage all the way to the right as shown below. Keeping the stage in this position, lift the belt off of the teeth on the optics platform base plate and shift it so that the spring is in the position shown. Reengage the belt on the teeth and install the belt clamp with the screw and washer shown.

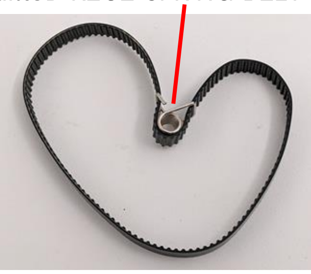

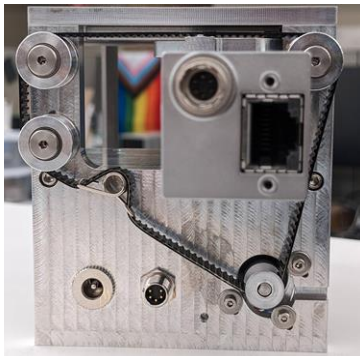

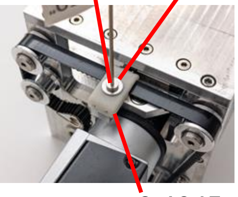

Push the motor fully back to the outermost position (you will encounter resistance from the spring) and tighten the mounting screws. Rotate the pulley so that the set screw is lined up with the slot in the plate as illustrated below. Adjust the axial position of the motor pulley to agree with the belt and the idler pulleys, then tighten the setscrew.

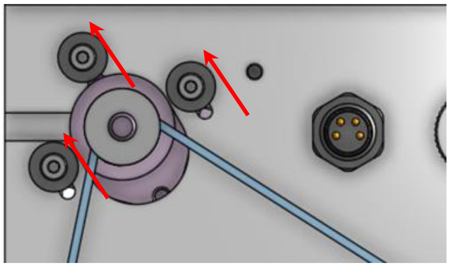

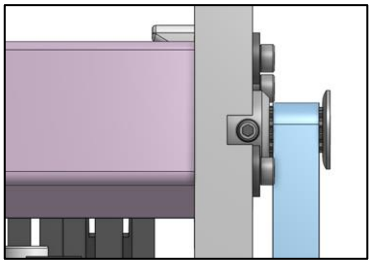

With the drive train now assembled, move the stage back and forth by hand from stop to stop to verify that it moves smoothly over the range of travel. Also verify that the homing switch activates slightly before the stage reaches the hard stop, and adjust the flag on the optics platform if necessary.

Install focuser and illuminator

Fit the focus locking sleeve assembled earlier onto the top tube on the turret and rotate it so that the upward facing arrow mark is on the same side as the printed markings on the mirror cube. Fasten it by *gently* tightening the three bottom set screws arranged around the bottom, just until the sleeve is immobilized.

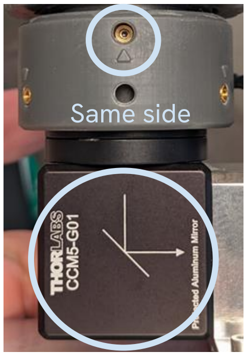

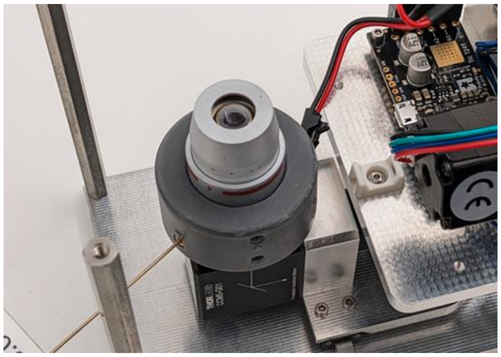

Check whether the objective can rotate freely. Overtightening the set screws that hold the collar onto the focusing tube will distort the tube and cause the threads to bind up. We have also found in multiple cases that the RMS-to-SM1 adapter ring on the objective seems to run slightly eccentric in the tube which causes the objective to interfere with the focus locking sleeve. If the latter is the case, it may be necessary to bore out the opening slightly to compensate for the eccentricity with extra clearance.

Install the focus knob by simply pressing it over the objective with the raised boss facing up as shown. The top of the boss should be slightly below the step on the objective.

Place the illuminator assembly built earlier onto the objective in the orientation shown below. It should rest on the step on the objective. Carefully secure the cable and header to the cable tie anchor with cable ties as shown. Plug the remaining connector from the power input harness into the header. Tighten the setscrew in the illuminator collar to secure it to the objective by inserting the hex key through the small hole in the shroud. If it is difficult to blindly align the hex key with the set screw this way, remove the shroud to access the set screw.

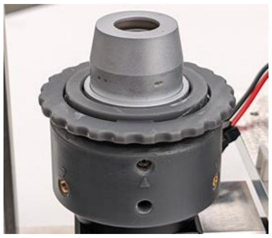

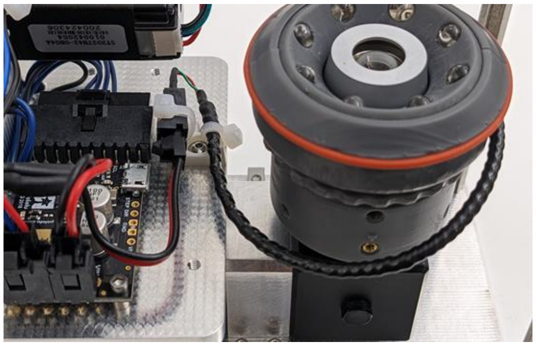

Top plate

Install the two Heli-Coil inserts (McMaster-Carr 96246A046) at the indicated locations from the top of the frame top plate (3-1241) if this operation was not ordered as part of the manufacturing of the part.

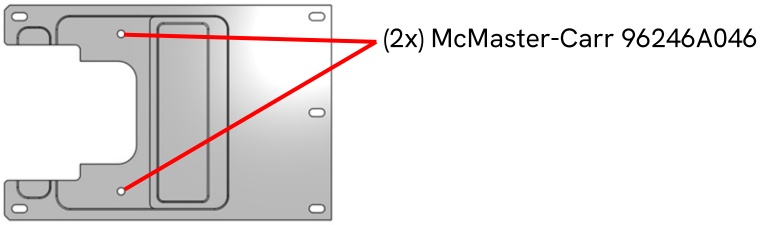

Place the top plate on the instrument and loosely install the 5 mounting screws and washers as indicated below. You will fasten the top plate after aligning it using the camera in a later step.

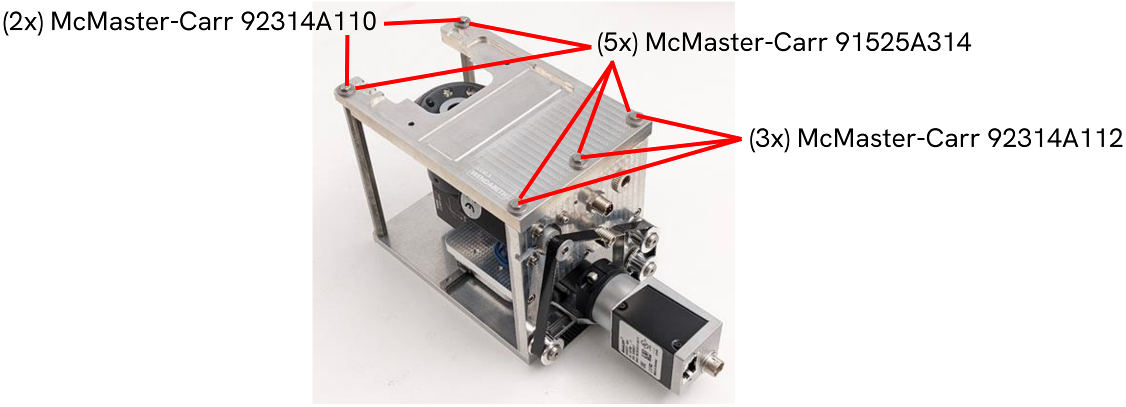

Commissioning

Software setup

The Basler Pylon Camera Software Suite is not a strict prerequisite to run the Cell Counting Imager software, but it includes a camera viewer tool which is useful for testing and adjustment, so it would be useful to install this first.
https://www.baslerweb.com/en-us/downloads/software/

Note! This document covers installation and usage of the CCI software at a surface level in order to support initial commissioning of the CCI. The primary practical documentation for the CCI software is packaged with the source code to ensure it is up to date as the software evolves.

The CCI software and documentation can be obtained from the GitHub repository here:
https://github.com/czbiohub-sf/accs-cell-counting-imager-pub

The Cell Counting Imager software is a Python package that should be installed in a dedicated Python virtual environment. How you choose to do this may depend on your platform, preferences and existing practices. For example, we use Anaconda so the process to install the CCI software from scratch would look like this:

```
cd /dir/where/you/downloaded/it/cell-counting-imager-0.2.0/
conda create -n accs-cci python=3.12
conda activate accs-cci
pip install .
```

Refer to the CCI software documentation for further information.

Basic stage function test

Connect the 24V power supply to the CCI. The LEDs on the illuminator should appear to light with a dull red glow and there should be some indicator light activity from the TIC board. The stepper motor should immediately energize and start holding the stage in place, which you can confirm by gently pushing on the stage.

Connect the USB serial cable to the CCI and then to the PC. Once you have the CCI software installed, you can run the included cci_test command,

cci_test --stage-auto

which will attempt to find which port the stage is plugged into and then run a movement test routine to make sure the stage functions correctly. If there is more than one FT232R based USB serial adapter connected then the program will list them for you and exit. If you are using a different type of cable or detection fails for some reason, you will need to check manually what port name your operating system assigned the virtual port to. You can specify the port to run the movement test with this variant of the command:

cci_test --stage COM4

You can also verify that the software can communicate with the camera with the following command, which simply captures an image and saves it in a JPEG file:

cci_test --camera

See the CCI software documentation for further details.

Using the Pylon camera viewer

As part of the CCI software setup process you will have installed the Basler Pylon Camera Software Suite, which includes the Pylon Viewer application. Review the online documentation to become familiar with how to operate the software:
https://docs.baslerweb.com/overview-of-the-pylon-viewer

Connect the CCI camera to the gigabit ethernet port on the PC via the PoE injector (TP-Link TL-PoE160S). Run Pylon Viewer, connect to the camera, and apply the following settings:

Pixel format	Mono12
Auto gain	Off
Raw gain	0
Gamma	Off
Binning factor, horizontal	2
Binning factor, vertical	2
Binning mode	Sum
Exposure time	120 ms

Enter Continuous Shot mode and you should see an image from the camera, which can be used for focusing and position adjustments as described in the following subsections.

Focus adjustment

If an actual flow cell and a sample of cells is not available, a convenient target for rough focus adjustment is an unassembled flow cell bottom plate (3-0305) or simply a piece of 1.5mm thick PMMA cut to the same shape without fiducials, with some fine scratches on the top surface.

To adjust the focus, loosen the setscrew in the illuminator collar until it can rotate freely, then loosen the upper setscrew in the focus locking collar and use the knob to rotate the objective. Note that this system is not very precise and the act of re-tightening the set screw tends to cause a significant shift in the focus. One can compensate somewhat by applying a counter-offset before tightening the screw and/or continuing to make adjustments while tightening the screw in small increments.

Top plate position adjustment

Mount a flow cell to the top plate, position the stage so that the fiducial mark in the first lane is vertically centered in the image, and adjust the alignment of the top plate so that the mark is not vignetted. Check against the fiducial in the last lane as well; if the fiducial is not in the same place in the X-axis then the top plate is not aligned squarely with the axis of travel of the stage.

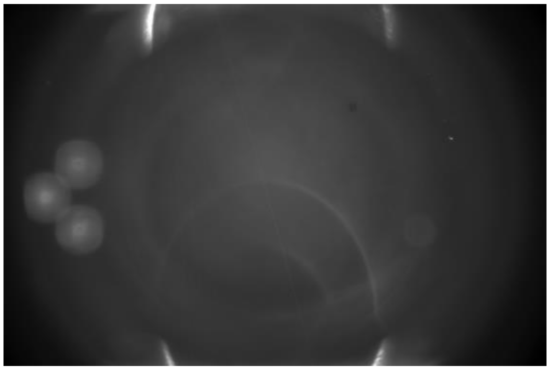

Once the result seems satisfactory, tighten the mounting screws to secure the top plate. Continue to observe the camera view while doing so as tightening the screws will likely affect the alignment.

Software stage alignment

The CCI software uses a configuration file to remember what positions to move to on a given instrument. Normally this only needs to be set up once for a given instrument unless something is changed mechanically or there is significant manufacturing variation in the lane positions between batches of flow cells or between individual flow cells.

Install a flow cell on the CCI, plug the USB serial cable into the instrument and the PC, connect power to the CCI, and follow the directions in the CCI software documentation to measure the lane positions and create the configuration file.

CCI flow cell design and fabrication

Overview

An exploded view of the CCI flow cell is shown below.

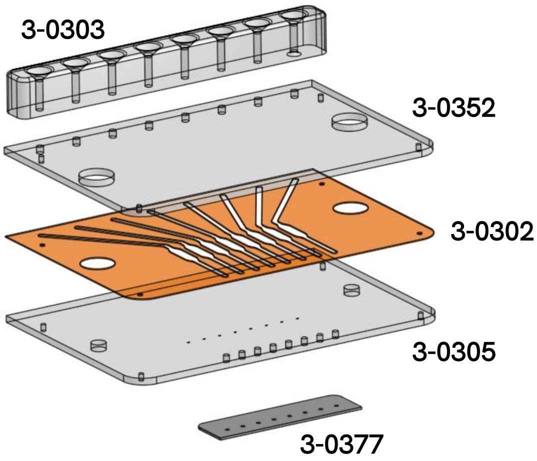

The core of the flow cell is a pair of PMMA plates (top plate, 3-0352; bottom plate, 3-0305) sandwiching an adhesive spacer layer (3-0302) which creates 8 fluid channels. A soft elastomer part on the top of the flow cell with chamfered openings (3-0303) forms a seal with the robot's pipette tips for injection of liquid. An etched PTFE strip with small exit holes covers the waste ports on the bottom of the flow cell and allows fluid to be pushed out by the pipette but inhibits wicking of fluid out of the channels which otherwise happens when the area around the waste ports becomes wet.

The flow cell was originally designed to be possible to fabricate without specialized equipment beyond a CO2 laser cutter. Files used for cutting the flat parts can be found as attachments to

the Cell Counting Imager Onshape document. The pipette tip interface was originally designed for "DIY" manufacturing as well but this was abandoned as discussed below.

Top (3-0352) and bottom (3-0305) plates

The top and bottom plates are cut from 1.5mm-thick cell cast PMMA material. The bottom plate has a set of etched fiducial marks which are used for image registration and for recognizing the center of the channel during manual calibration of stage positions.

Spacer layer (3-0302)

The "in house" version of the spacer layer was cut from Grace Bio-Labs SecureSeal adhesive sheet, 0.24mm thickness (SA-S-2L) using a CO2 laser cutter.

Pipette tip interface (3-0303)

The pipette tip interface is cast from a Shore 40A hardness, water-clear polyurethane rubber material by an online prototype manufacturing service.

The pipette tip interface was originally produced in-house by casting PDMS in a custom mold made of machined aluminum plates and dowel pins but this was eventually abandoned due to the constraints it posed on the design of the part and the unreasonable amount of effort and mess involved required to produce the part this way compared to simply ordering elastomer castings from a prototype manufacturing service based on a solid model file.

The pipette tip interface is bonded to the flow cell using an adhesive gasket made from the same material as the spacer layer.

Waste nozzle strip (3-0377)

The waste nozzle strip is produced from 0.02" thick, adhesive-backed PTFE sheet (McMaster-Carr) by etching an extremely fine checkerboard pattern before cutting out the drain holes and the outline of the part.

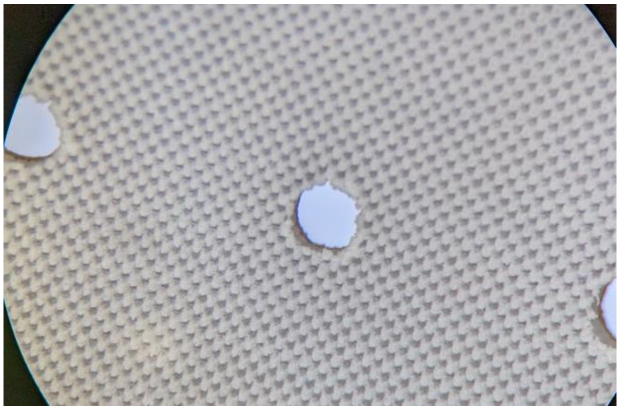

The combination of the small exit holes and the superhydrophobic laser-treated surface surrounding them are designed to force exiting liquid to break away and fall in small drops rather than wet the surface and form a wicking path into the waste trough or large hanging drops that pull liquid out of the channels.

General operating procedures

This section contains a few key points about general handling of the instrument and flow cells. Users will need to develop their own detailed SOPs as appropriate to their situation.

Mounting consumables

The waste trough and flow cell are mounted on the CCI as illustrated below. The waste trough must be placed first as the flow cell blocks it from being removed. Both items can be left in place for multiple experiments if the waste trough is emptied by aspiration as necessary. After use the consumables should be removed to allow removal of any spilled liquid that may have seeped into gaps, and all items should be cleaned as described below.

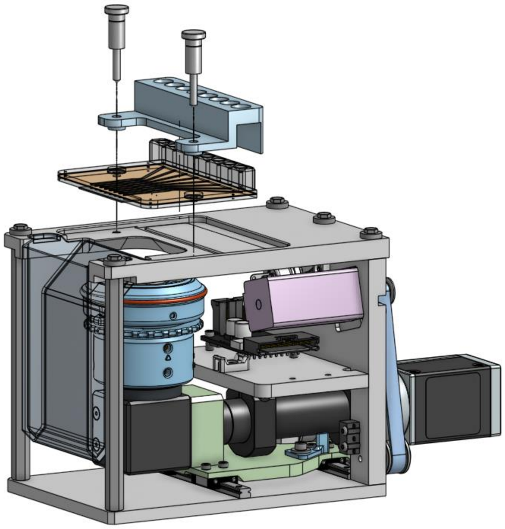

Automated operation

The CCI is normally mounted on the deck of a pipetting robot and controlled using simple HTTP requests. In the context of ACCS these requests come from the protocol script running on the robot. Refer to the CCI software documentation for more details.

Information pertaining to use of the CCI from the operator's perspective, in the context of an automated cell culture workflow, can be found in the *CCI Normalization Sample SOP*, available separately as a supplement to the ACCS manuscript.

Empirical calibration

Currently the CCI software uses a single calibration factor, supplied as part of the configuration file, to convert cell counts to concentration. This allows us to account for a variety of non-ideal factors (software counting sensitivity, manufacturing variation of the flow cells and the instrument itself, light source aging, etc.). Because of the relatively frequent turnover of flow cells and the labor involved in making a quality ground truth measurement, we do not re-calibrate for each individual flow cell, and the software currently does not use separate calibration values for each channel on the flow cell (though adding support for this would be relatively straightforward). In practice we find that any systematic inter-channel variation in readings for a given flow cell (e.g. due to variations in material thickness, channel geometry, reflective debris, etc) is generally obscured by other sources of noise and bias.

Our typical procedure to calibrate the CCI is to prepare linear dilutions of stocks of the relevant cell type, spanning the expected concentration range, then use repeatedly measure these with the CCI (with the help of the OT-2) as well as with a reference instrument, and use a

linear regression to generate the calibration factor. For full details, refer to the procedure described in the ACCS manuscript and/or the Supplementary Materials and Methods, as well as the analysis code provided in the main repository for the publication. Of course, for other use cases, samples could be loaded manually and the CCI scans performed with (e.g.) a simple Python script.

The CCI software distribution includes an example protocol script for the Opentrons OT-2 to load a series of samples for CCI calibration measurements. The default CCI configuration also includes a generic calibration factor that should be "close enough" for basic use if a reliable reference instrument or appropriate cell stock is not available for calibration purposes.

Quality checks

When ACCS is being used in production, we perform a weekly Monday morning quality check by preparing a cell dilution with a particular nominal density (e.g. 250k cells/mL) and running a short OT-2 script that loads the same stock and takes measurements with the CCI several times in a row. The stock is prepared by dissociating HEK293T cells grown over the weekend, taking a concentration measurement with a Countess II cell counter, and diluting accordingly. We plot the resulting data and flag any statistical anomalies based on arbitrary alert thresholds, e.g. any outliers not explained by air bubbles picked up by the pipette, >12% CV between channels on a given scan, >15% CV across cumulative readings from all 6 measurements, or an average measured concentration further than ±25% from the expected value.

The CCI software distribution includes an example protocol script to run the aforementioned QC measurement protocol.

Flow cell storage and working life

No special storage conditions are observed for new, unused flow cells or flow cell components.

Once a flow cell has been exposed to biological materials it is kept in a dated plastic container (e.g. a 150mm round culture dish) under refrigeration. Used flow cells are discarded after at most 31 calendar days as a proactive contamination control measure. We therefore do not have an empirical basis to predict a practical maximum service life for a CCI flow cell.

Flow cell cleaning

The exterior surfaces are wet cleaned before and after use with a commercial plastic cleaning product (Blue Ribbon Plexi-Clean) and soft polyester cleanroom wipes (Texsorb Absorbond TX404). Exposing the PMMA surfaces to alcohols should be avoided as it induces stresses in the material and can cause crazing under some conditions.

Cleaning of the internal channels is an automated process built into the ACCS protocols. The robot fills the flow cell with a strong detergent solution (Decon Laboratories Contrad 70, diluted to 10% v/v in DI water) and soaks it for 60 seconds, then flushes three times with 200 µL of water.

Cleaning the CCI instrument

The open-frame design of the CCI poses some limitations on cleaning methods. It should not be sprayed down with liquid. Exposure to incidental mists of alcohol solution while cleaning the biosafety cabinet and robot deck is likely to be harmless.

We normally soak a laboratory wipe in 70% ethanol and spot clean the top of the instrument as well as any surfaces exposed to drips or spills. Cleaning the linear guide rails should be avoided if possible. In the case of a spill reaching the guide bearings, proactive replacement of the affected linear guide should be considered as there is not a practical way to decontaminate the internals and it may eventually start to fail.

The waste trough is normally disinfected with a 10% bleach solution. The trough should be emptied of bleach solution and allowed to dry before mounting it back on the CCI.

Supplement D.
Sample CCI Normalization SOP

EXAMPLE

ACCS CCI Normalization SOP

Uses Opentrons software version 4.7, protocol framework `t220929a` or later and the CCI2.5 hardware.
DO NOT ACCEPT ANY DIALOG OFFERING TO UPGRADE THE OPENTRONS APP OR ROBOT SOFTWARE.

Protocol Description:
This protocol takes a 96 well source plate containing adherent cells and splits it out to a propagation plate, using the CCI to normalize to specified seeding targets (cells/well) for each well. Optionally it then distributes cells to a second plate at the same density (but optionally a different amount). Each destination plate can be either a standard 360uL 96-well plate or a 440uL glass-bottom imaging plate. By default a single plate will be filled to 200uL or two plates to 150uL each.

1. Initial prep
    a. Start thawing/warming up reagents to 37°C
        i. At least 8mL of 0.25% trypsin-EDTA dissociation reagent
        ii. At least 60mL of suitable growth medium including 10% FBS and 25mM HEPES
    b. Open BSC sash and start fan
    c. Wipe down surfaces in the BSC
    d. Take a dish with a flow cell from the refrigerator and move it to the BSC (it needs a while to warm up to avoid condensation)
    e. Install tip trash liner:
        i. Cut new bag to half height and discard upper half
        ii. Tuck bag into bin and secure with binder clips (you can take the bin off the deck to make this easier). Make the bag as flat as possible against the floor and inside walls of the bin, especially around the front-left corner. Make sure the overhanging plastic on the outside is kept neat and does not encroach over neighboring deck slots (see example below).

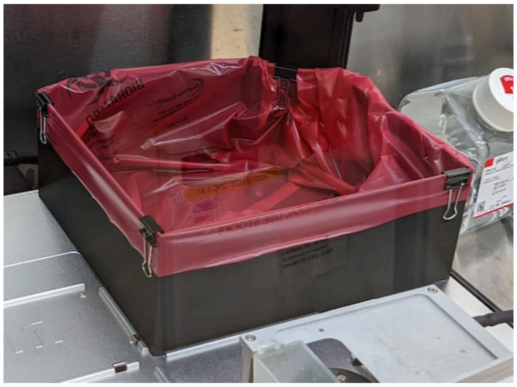

        iii. Put tip trash bin back in place on robot deck

EXAMPLE

2. <u>CCI Prep:</u>
    a. If the CCI is not in place already, install it on Slot 6 of the OT2, with the waste cup on the left side and the connectors on the right.
    b. Make sure the three cables to the CCI are plugged in as shown
    c. Make sure the ethernet cable plugged into the camera is not obstructed by the other cables and has room to move back and forth

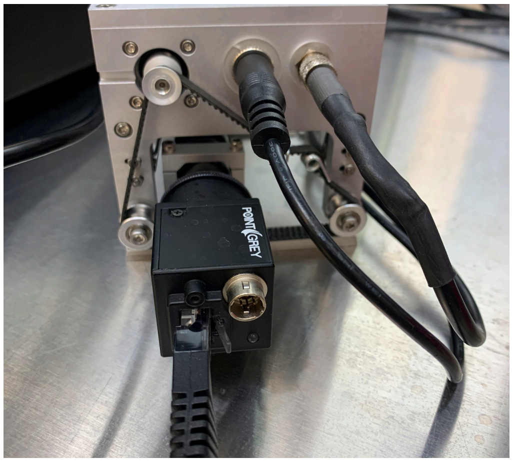

3. <u>Flow cell prep:</u>
    a. If you are using a brand new flow cell, make sure it has received the Pluronic F-127 pre-treatment as described in the Cell Counting Imager Technical Manual.
    b. Check the flow cell for debris and/or residue on both sides, particularly over the wide part of each channel (where imaging takes place).
        i. Clean with Plexi-Clean and a lint-free cloth as necessary. Do not use alcohol.
        ii. Use compressed air as necessary to blow off any dust and/or lint left after wet cleaning.
        iii. If the flow cell is still cold from the refrigerator it may form condensation. Defog the surface with a gentle stream of compressed air if necessary.
    c. Put the flow cell in place on the CCI
        i. Handle the flow cell by the edges as much as possible and avoid touching the top and bottom surfaces near the imaging area
        ii. Place the flow cell with the mounting holes lined up with the holes on the CCI deck and drop it into place.
        iii. Push the flow cell to the left (towards the waste trough) to make sure the rounded end is pushed up against the wall.
        Push from the mounting holes (again, avoid touching the imaging area).
        iv. Do not install the tip guide and thumbscrews yet!
    d. Clear the flow cell channels of liquid
        i. Place a cloth over the opening of the waste cup on the left side of the CCI to mitigate flying droplets
        ii. Line the nozzle of the compressed air gun up to each of the holes on the clear rubber tip interface on the flow cell and apply just enough flow to blow out any liquid from storage or the previous run. A slight fog or micro-droplets may remain on the inside surfaces.

4. Robot startup
    a. Turn on the robot, tempdeck, CCI camera and CCI stage using their designated switches on the outside of the BSC.
    b. If the robot was not turned on already, it will take a few minutes to start up. When the robot is ready the blue light will stop flashing and you will a few mechanical noises.
    c. Open the Opentrons app. Connect to the robot by clicking on the toggle next to the relevant robot name (e.g. OT2-SWEETPEA). Make sure you select the correct robot! Each robot has a name label attached on the front beneath the logo.

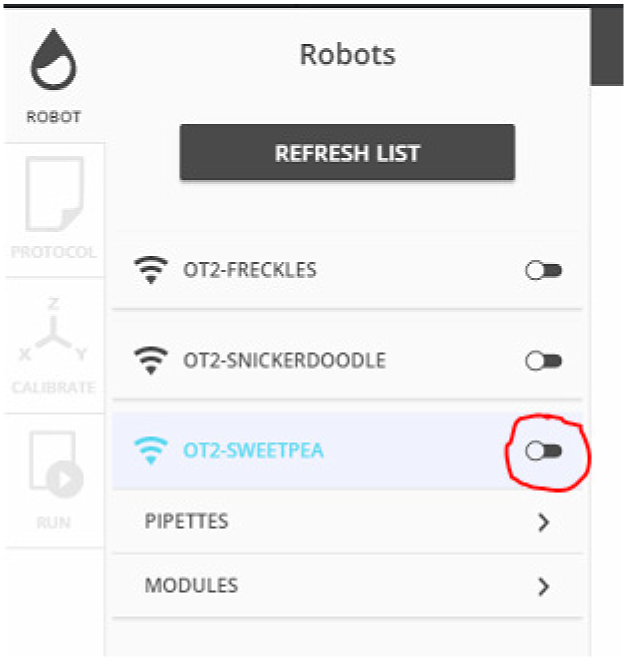

EXAMPLE

5. Prepare the protocol script
   a. Open the "Splitting software" folder on the desktop, then "Protocol setup" and then finally run "Setup CCI Normalization (NPF)". A browser window should pop up and show a Jupyter notebook called "CCI Normalization Protocol Setup".
   b. Click the ⏩ button as instructed to initialize the form
   c. Fill out the form fields
   d. Click the "Generate script" button and the path to the generated protocol script should appear along with a confirmation message.

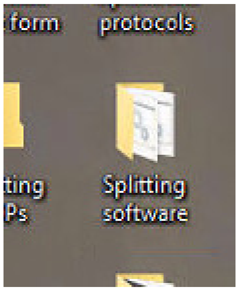

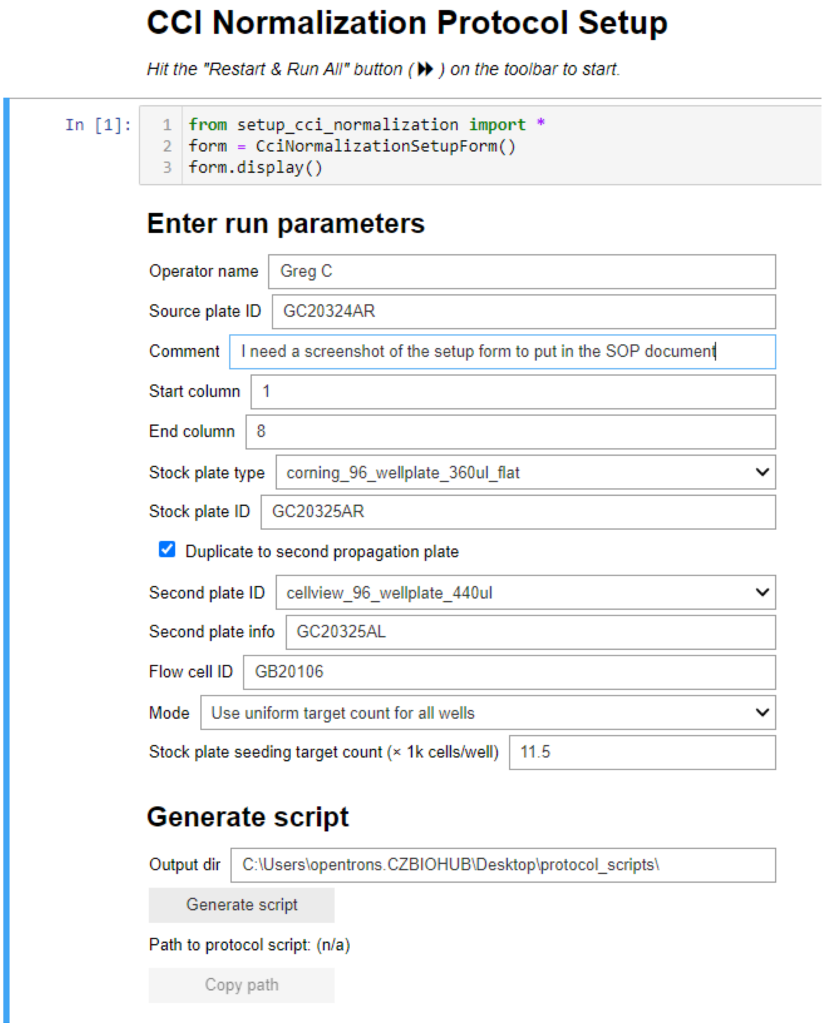

EXAMPLE

6. <u>(if needed) Labware calibration</u>
   This does not need to be done for every run! Skip this section unless the robot has been moved or there is some other reason to believe the calibration needs to be checked/adjusted.

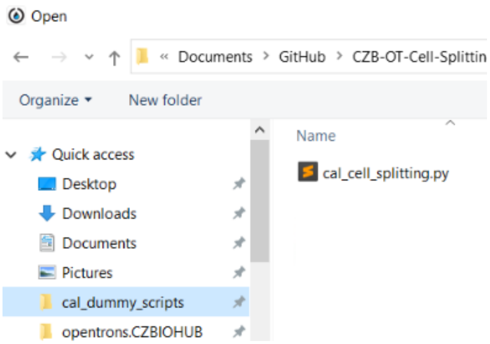

   a. In the Opentrons app, open the protocol called "`cal_cell_splitting.py`". You can find it in the dialog via the "cal_dummy_scripts" shortcut in the Quick Access pane.
   b. Select the "Calibration" tab in the app, ensure the tempdeck is powered up, and click "Continue to labware setup" if shown
   c. Set up labware for calibration
      i. Refer to the deck layout on the "Calibration" tab of the Opentrons app and install labware as applicable.
         - See the "Tip for installing labware" inset below this section if you are unfamiliar with attaching labware to the OT-2 deck.
         - You can use spare / nonsterile labware (96 well plates and 12-well reservoir) for the calibration process.
         - Only install the labware types you are actually using (i.e. if you are not using a microscopy plate, you don't need to put anything in slot 1).
         - Put each applicable type of labware in the specific location indicated, regardless of what arrangement of plates you are using for your actual run
         - Do not place your source plate on the tempdeck yet.

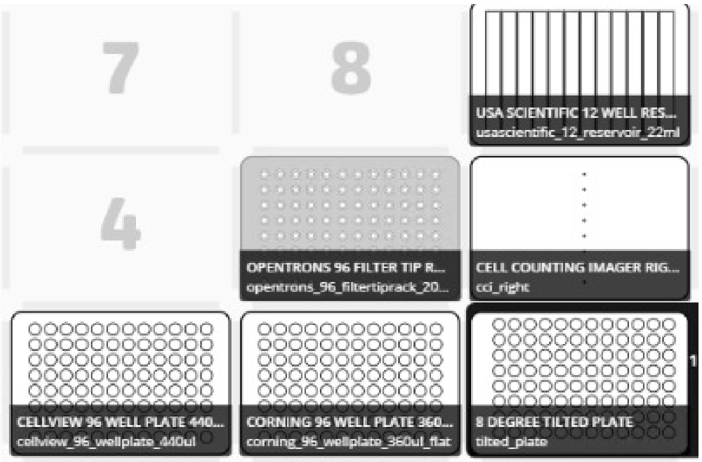

      ii. Install a box of tips on slot 5 for calibration. You only need the leftmost column filled with tips. It is recommended to use a leftover open box for this.
      iii. Install the tilted plate calibration tool on the tempdeck.

EXAMPLE

d. Review the following notes, then follow the steps on the Calibration tab of the Opentrons app to proceed through labware calibration.
Note: After the tip box is done, you can choose the order to calibrate labware in by manually selecting from the deck diagram instead of using the "next" button. It may be beneficial to go in the order listed below.
   i. Tip box (slot 5)
      - Do not change the calibration unless the alignment is visibly off and/or tip pickup fails. Pick up tips, save, and continue.
   ii. Trough (slot 9):
      - Make sure the tips are roughly centered over the first well of the reservoir. The ends should be level with the top surface of the labware and not below it.
   iii. CCI (slot 6):
      - Make sure the flow cell is seated over the window on the CCI.
        The black tip guide should not be in place yet!
      - The ends of the tips should be (on average) centered in the entry cones and should dip about 1/8" below the top surface of the pipette tip interface, down to the bottom of the entry cones where the narrow channels start.
   iv. Tilted plate (slot 3)
      - Verify that the tilted plate calibration tool is in place on the heat block before sending the robot to that slot.
      - Z position should be such that the ends of the tips are in plane with the top surface of the tool or a little above -- verify by looking with your eyes level with the top of the tool (see photo). Ensure the tips are not down inside the groove at all; err on the "too high" side.

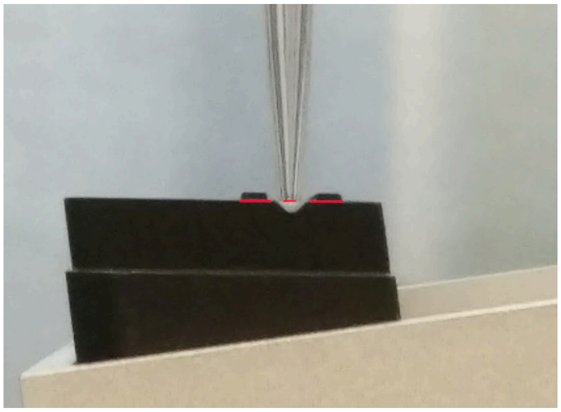

      - X and Y position should be such that the tips *(on average)* line up with the raised crosshairs. It will never be perfect; it is normal for all 8 tips to be *slightly* off in different directions.

EXAMPLE

v. Destination plate(s) (slots 1 and 2)
   - You will probably want to use an identical spare plate for calibration instead of your sterile propagation plate
   - Make sure there is no lid on the plate before sending the robot to that slot
   - If you will not be using the type of plate assigned to a given slot, you can just omit the plate, accept the calibration and move on without changing it.
   - Center the tips on the wells, making sure the ends are level with the top rims and not poking down into the wells.

e. After you have calibrated every item, click "Return tips and proceed to run" (but do not actually proceed to run yet).

f. Remove the tilted plate calibration tool and any non-sterile labware used for calibration. Wipe down the deck again if appropriate before proceeding to install your sterile labware.

Tip for installing labware

1. Put the front right corner of the labware against the deck
2. Push it towards you and to the right until it fully compresses the spring clips

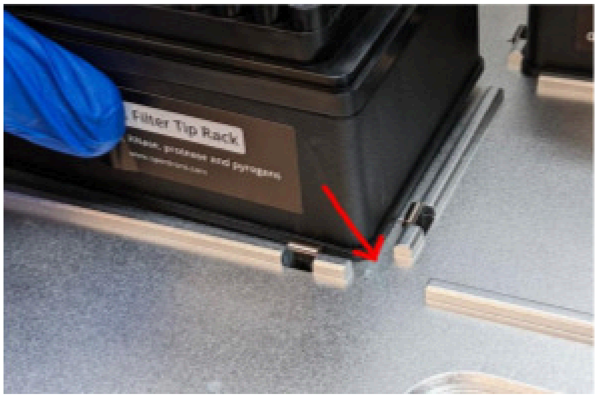

3. While holding the labware against the clips, push it down flat to seat it
4. Release and visually confirm proper position. Rails should be visible on all 4 sides.

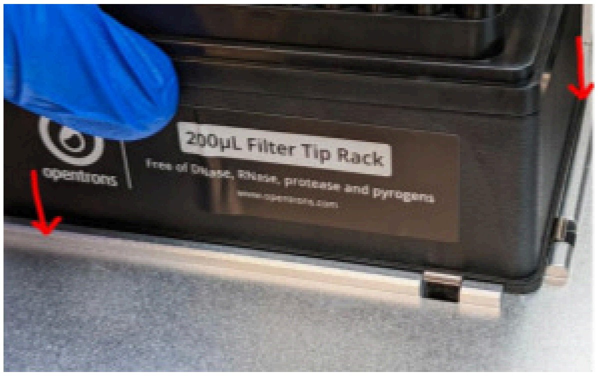

EXAMPLE

7. Set Up
    a. Go to the Run tab in the app and set the tempdeck to 41°C so it has time to stabilize
    b. In the Opentrons app, open the protocol script you generated in the previous step. The simulation process will run while you complete the rest of the preparations.
    c. Load reagents in trough in the arrangement shown below. Consider adding the trypsin last.

1. Waste (empty)	2. Waste (empty)	3. PBS (20mL)	4. PBS (20mL)	5. Trypsin (8mL)	6. Media (20mL)	7. Media (20mL)	8. Media (20mL)	9. DI water (8mL)	10. Cleaning soln* (4mL)		

*10% Contrad 70 in distilled water. Provided in a tube labeled "CCI CLEANING SOLN".

    d. Ensure your source plate contains no more than 200µL of media per well. Remove some liquid if necessary.
    e. Finish loading labware on the deck.
        i. You can check the Calibration tab in the Opentrons app to determine how many tip boxes to install and where. Install remaining labware according to the layout below.
        ii. When mounting your source plate on the tempdeck, push down to make sure it is fully seated. The fit may be snug on some plates.
        iii. When loading tip boxes, go in order from right to left and back to front, making sure they are properly seated inside the rails, not overhanging or offset.

10<br>Tip box?	11<br>Tip box	(Trash)
7<br>Tip box?	8<br>Tip box?	9<br>Reagent trough
4<br>Tip box?	5<br>Tip box	6<br>CCI
1<br>Propagation plate 2?	2<br>Propagation plate 1	3<br>Source plate (on tempdeck)

8. CCI startup
   a. Install the pipette tip guide onto the flow cell and fasten it to the CCI with the two thumbscrews. Make sure to keep turning the thumbscrews until you feel a positive stop. It is important for the flow cell to be seated properly.
   b. Make sure there is not already a "CCI Server" console window open!
   c. From the "Cell Splitting" folder on the desktop, double-click on "Run CCI".
      i. A console window titled "CCI Server" will open. You should eventually see something like this:

```
======== Running on http://0.0.0.0:80 ========
(Press CTRL+C to quit)
```

      ii. If you see an error instead, seek assistance from a support contact before proceeding
   d. Turn off the light in the BSC

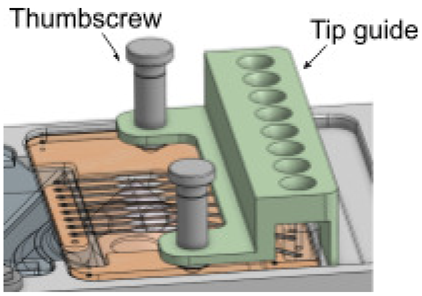

EXAMPLE

9. Final check

Always double-check before starting the robot:

  a. Did you remove the tilted plate calibration tool and install your source plate?
  b. Did you replace the labware used for calibration on slot(s) 1/2 with your actual sterile plate(s)?
  c. Are the tip boxes and trough aligned and seated properly in their slots?
  d. Are the lids off of all plates and tip boxes?
  e. Do you have the CCI software running?

10. Run the protocol
  a. Go to the Run tab in the Opentrons app and start the protocol
  b. Make sure you have joined the relevant Slack channel (e.g. `#ot2_error_sweetpea`) to receive notifications
  c. IMPORTANT:
     Check on the tip waste bin occasionally to make sure tips are not stacking up
     i. You can use an aspirating pipette or other convenient object to gently rake the tip pile to the rear and right sides of the bin, away from the "drop zone".
        Be very careful not to knock or launch tips out of the box in the process, and be aware of the robot's movements. It may be prudent to pause the robot.
  d. IMPORTANT:
     If you are splitting to two destination plates, you will need to replace the tip box on Slot 5 once all the tips in it have been used.
     i. Pause the protocol from the Opentrons app (it is recommended to wait until the robot goes to the trash to discard a tip). You can proceed without pausing the robot but this is at your own risk.
     ii. You may want to cover your destination plate(s) before reaching over them to swap the tip box
     iii. Make sure the new tip box is properly seated and you haven't bumped any neighboring boxes and unseated them

11. <u>Clean up</u>
    a. Bleach source plate
    b. Incubate propagation plate(s)
    c. Consolidate leftover tips in boxes marked "non-sterile" and save for future use. Recycle empty boxes and extra lids.
    d. Aspirate all liquid in the reagent trough and dispose of it
    e. Empty and clean the CCI waste cup
        i. You can free the waste cup from the CCI by lifting up and then sliding it out to the left
        ii. Aspirate all liquid contents
        iii. Disinfect by washing down the inside with 30% bleach solution and then aspirating the excess
    f. Put the flow cell away
        i. Undo the thumbscrews, remove the tip guide, then remove the flow cell from the CCI
        ii. Dab away any excess liquid on the outside of the flow cell with a Kimwipe, then place the flow cell back in its dish, upside-down with the orange drain gasket facing up.
        iii. Add a tally mark to the lid of the dish and place it back in the fridge.
    g. Clean up the CCI
        i. Remove any excess liquid on the top plate of the CCI, then wipe it down with a Kimwipe moistened with 70% ethanol. Avoid letting liquid get on the objective and do not spray anything directly onto/into the instrument.
        ii. Cover the CCI by placing a tipbox lid on top of it
    h. Unclip the liner from the trash bin and dispose of the used tips. Check around the work surface and robot deck for any loose tips.
    i. Disinfect the OT2 deck, trash bin and BSC work surface with 70% ethanol. Avoid spraying liquid on the CCI or into the air vents on the tempdeck.
    j. Disinfect the CCI tip guide, thumbscrews and trash liner clips with 70% ethanol and place back in the storage dish
    k. Shut the BSC sash
    l. Turn off the CCI, OT2 and tempdeck from the switches on the power strip outside the BSC

Supplement E.
Further investigation of effects on growth and viability

Abstract

Here we further explore potential impacts on cell viability and morphology from passaging cells using ACCS as compared to manual methods, including under extended incubation periods of up to 3 days. We seeded sets of plates with a fixed splitting ratio, both by hand and with the robot, then performed ATP viability assays at approximately 1, 2 and 3 days from seeding. For each condition we also captured 10X phase contrast images from preselected random sites on the plate before the terminal assay to provide examples of morphology. We also investigate possible impacts of the timing of seeding individual wells within a given ACCS passaging run.

Results and discussion

Viability over various incubation intervals

We measured total ATP to assess overall viability of plates seeded by the robot after incubation periods of 1, 2 and 3 days, as compared to plates seeded manually. For the robot-passaged plates we observe well-to-well variation similar to or better than their manually passaged counterparts and no detriment to viability attributable to handling by the robot.

Figure S3 illustrates CellTiter-Glo 2.0 assay results obtained from test plates at roughly +1, +2 and +3 days since seeding. The test plates were seeded with a fixed 1:6 split factor from nominally uniform source plates and cultured in parallel. Each point on the plot is the mean value over 76 test wells, except that 10 wells are excluded from the second manually-seeded plate in Fig. S3(A) due to the corresponding row on the hand-seeded source plate being inadvertently double-seeded. Figure S4 shows the distribution of scaled assay values from each read.

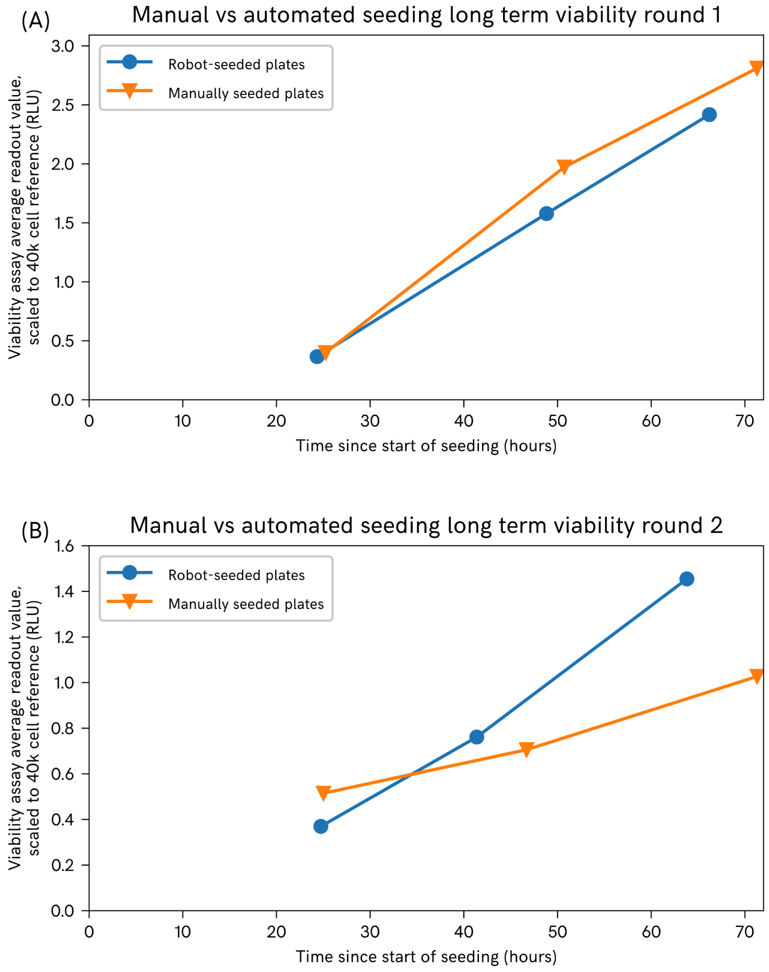

Fig. S3. Average of ATP assay readout values for each test plate, scaled to a reference sample on each plate with 40k cells/well, plotted against age of the plate since seeding. To be clear, the assay is terminal and each point represents a different plate. Panels **(A)** and **(B)** represent experiments carried out on separate weeks.

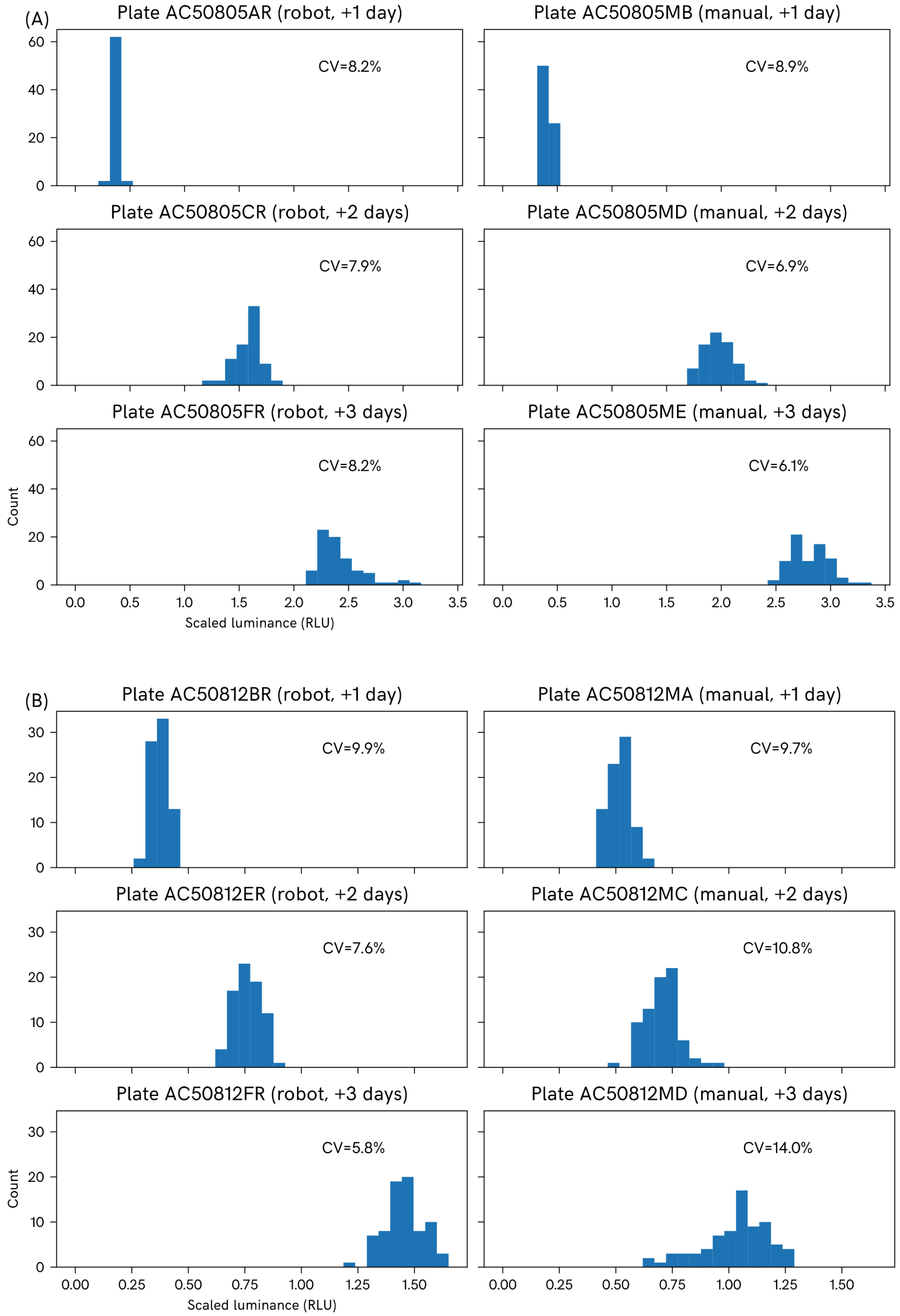

Fig. S4. Distribution of individual well values for each test plate. Subfigures (A) and (B) correspond to the data in **Fig. S3(A)** ("round 1") and **S3(B)** ("round 2") respectively.

Effects of seeding order

Using the same datasets we also investigated whether the order wells are seeded in by the robot affects recovery and growth (wells seeded earlier in the process). We used the ACCS protocol logs to determine the time when each well on a plate was seeded and analyzed the assay scores against well seeding time.

As illustrated in Figure S5, on 5 of the 6 plates we observe a possible positive trend in viability scores with respect to the time the well was seeded. However, the effect is very small compared to other, uncorrelated variation in the data, and all but disappears at +2 days and beyond (in fact, in one case the trend is actually slightly negative).

Assuming we are not being confounded by some other, downstream phenomenon correlated to position on the plate, we speculate that such an effect could be a consequence of cells being subjected to varying lengths of ambient exposure immediately post-seeding according to their position on the plate and the order the wells are seeded in.

In our use cases we find a systematic effect of this magnitude is of virtually no practical significance and makes up a small fraction of the overall error. However, we also note that it would be straightforward to decouple seeding time from well location by adding a randomization strategy to the protocol script if desired. Further, any effect found to be due to temperature stress could be mitigated by adding a second heat block to keep the destination plate contents at an appropriate temperature throughout the protocol.

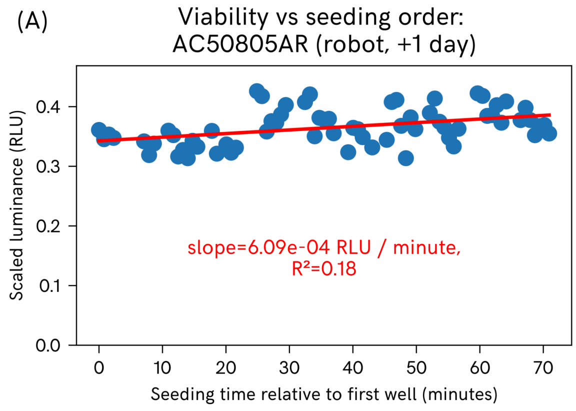

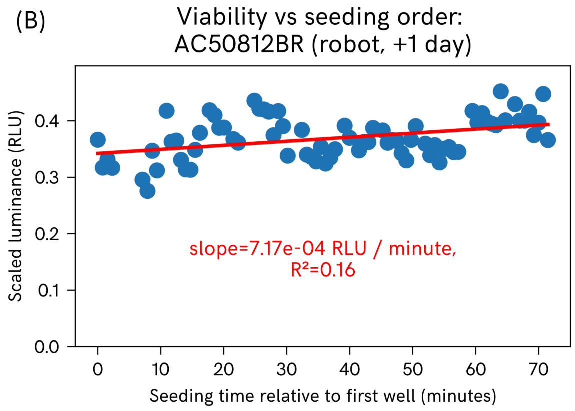

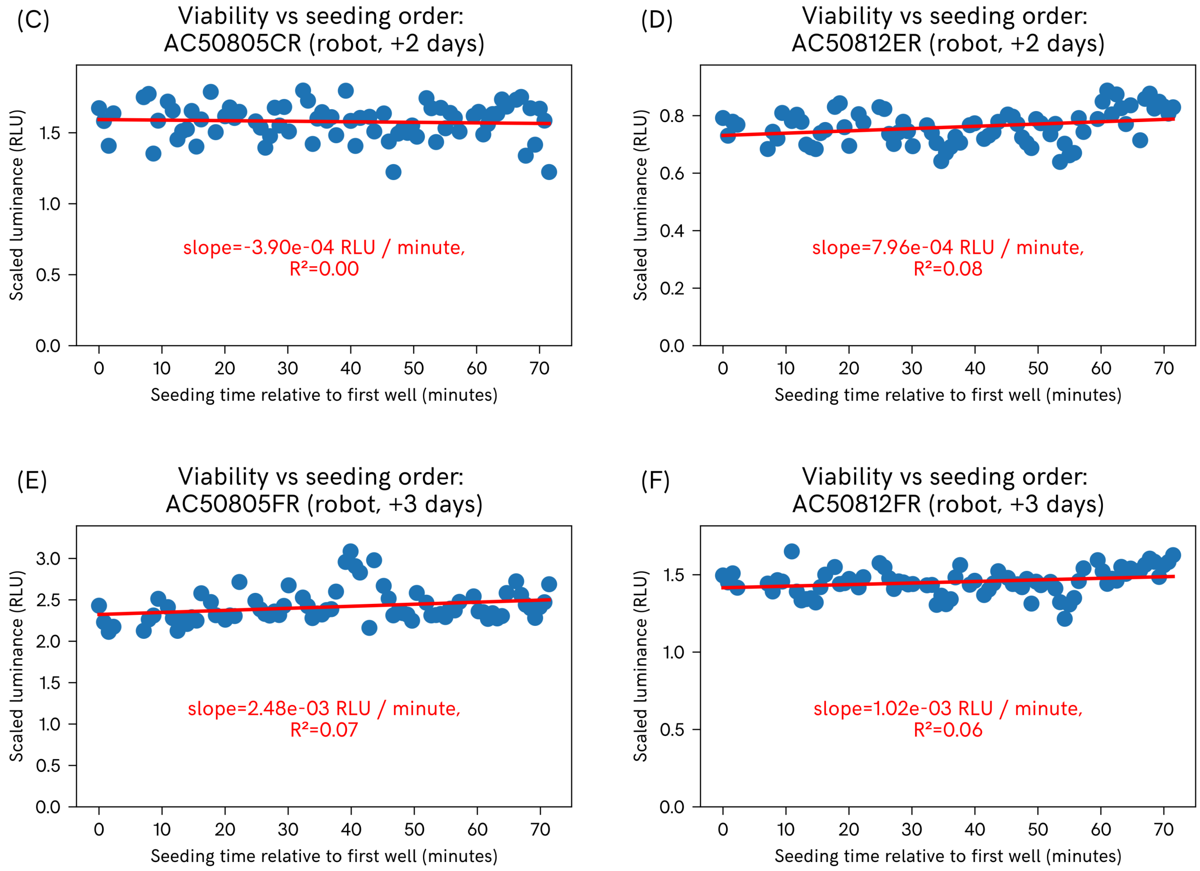

Fig. S5. Viability assay results plotted with respect to relative point in time when each well was seeded, with fit lines obtained by linear regression. Assay values are scaled to a reference sample on each plate with a suspension of 40k cells / well.

Morphology images

This section presents a set of representative morphology images taken at 10X. The location references and imaging procedure are explained in the Materials and Methods section.
Overall we observe healthy cell growth and no systematic abnormalities in the robot-passaged plates compared to the manually-passaged plates.
Full size images and original raw files are available to download from the public repository:
https://github.com/czbiohub-sf/2024-accs-pub/accs24-pub-aux-data/supplement_e/morphology_pics.

1-day-old plate

	Robot-split plate	Manually split plate

B2<br>upper<br>middle	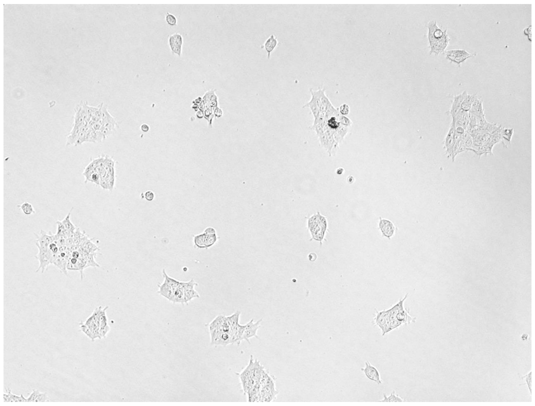	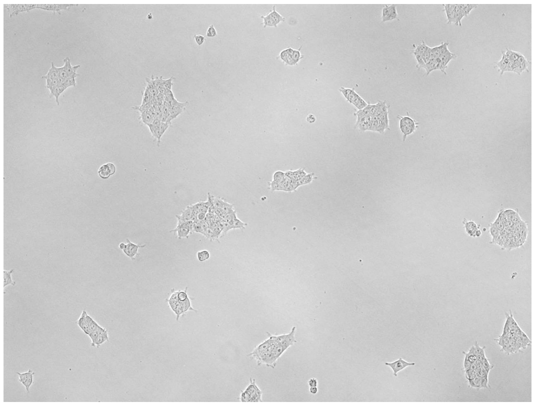
C9<br>lower<br>middle	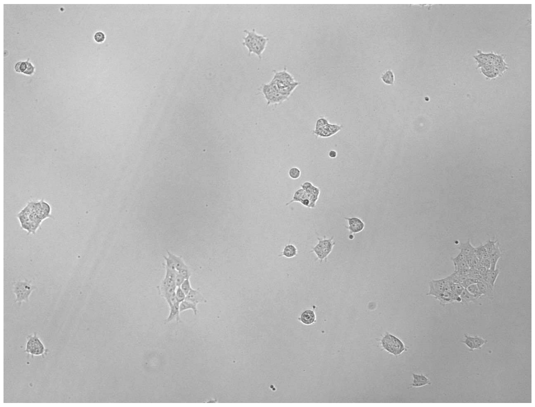	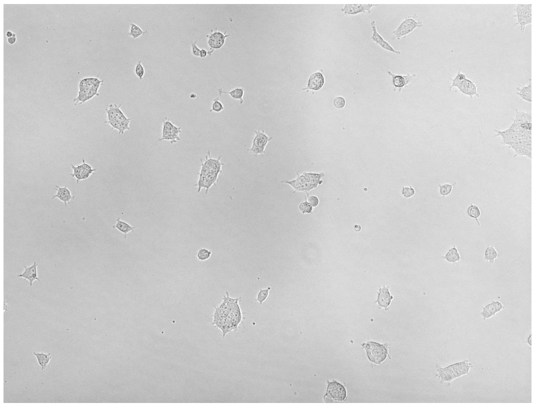
E8<br>lower<br>right	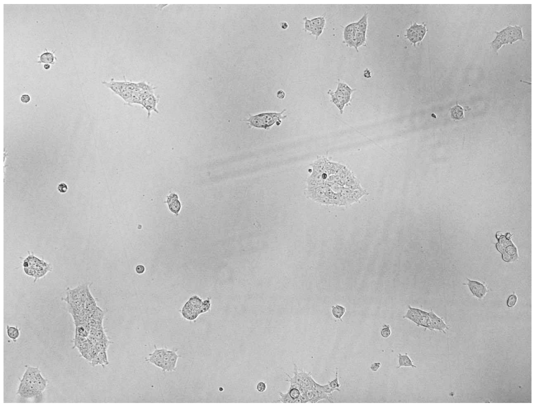	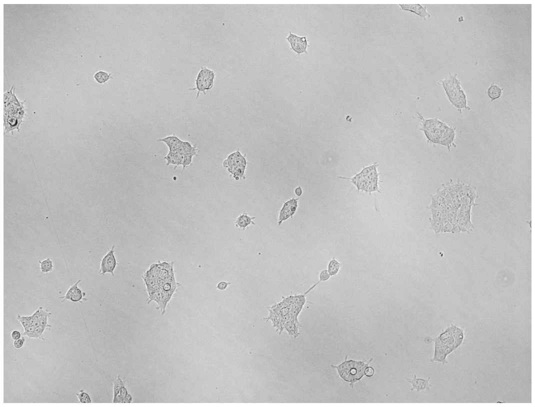

F8 right middle	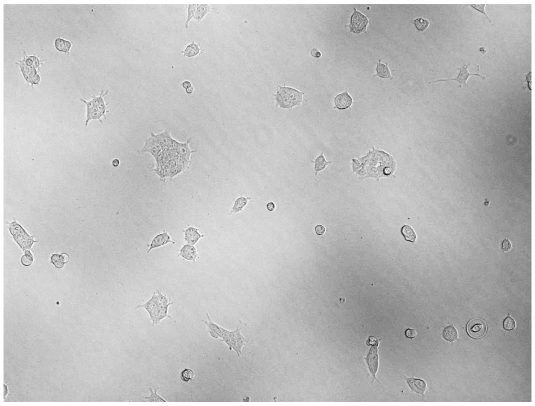	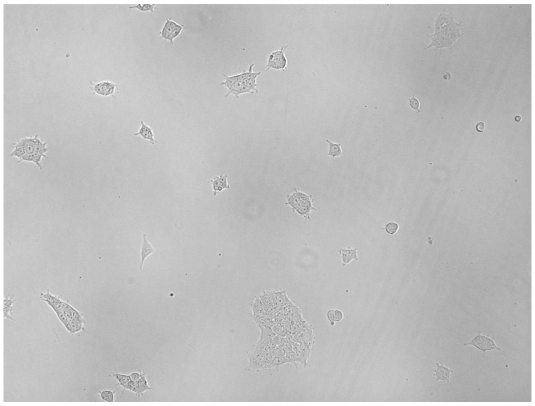
G3 center	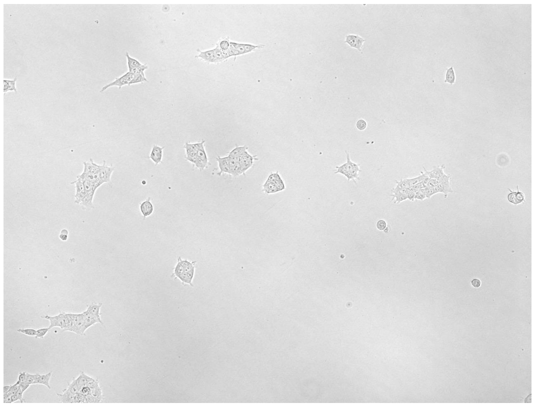	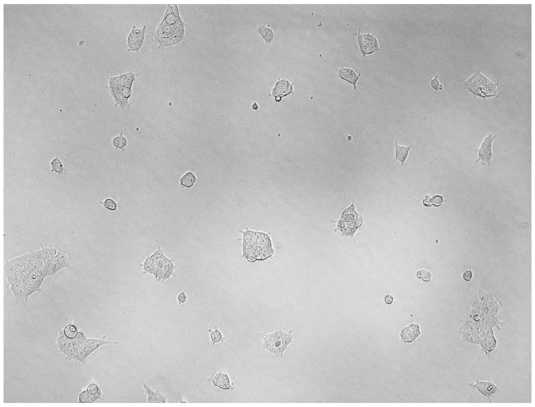
G4 upper left	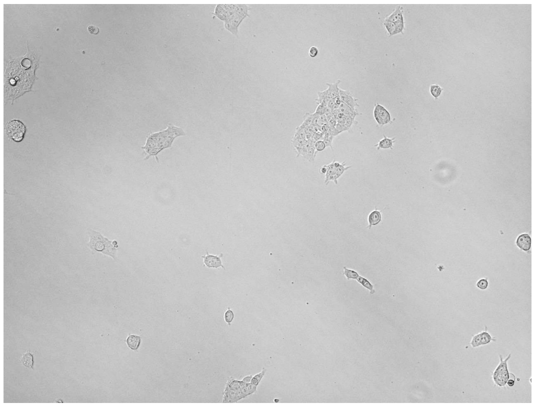	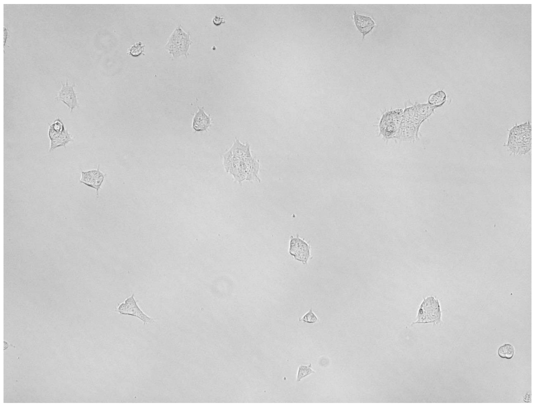

2-day-old plate

	Robot-split plate	Manually split plate

B2<br>upper<br>middle	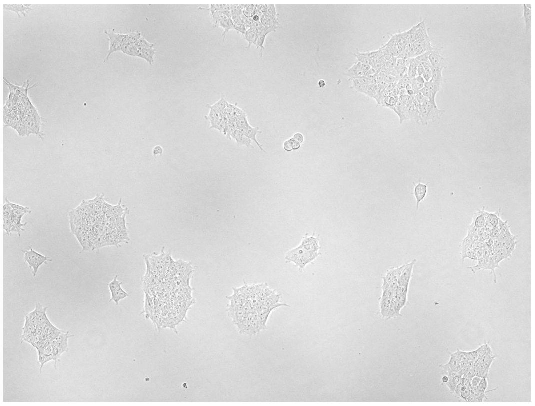	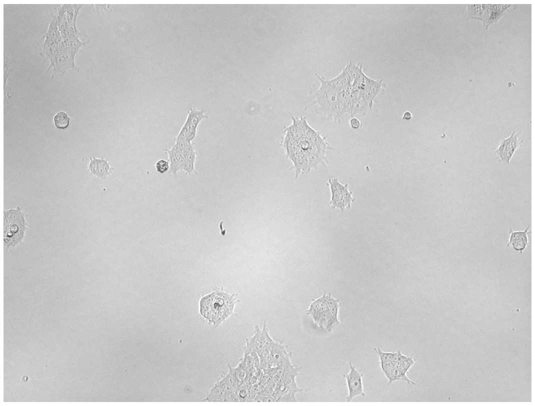
C9<br>lower<br>middle	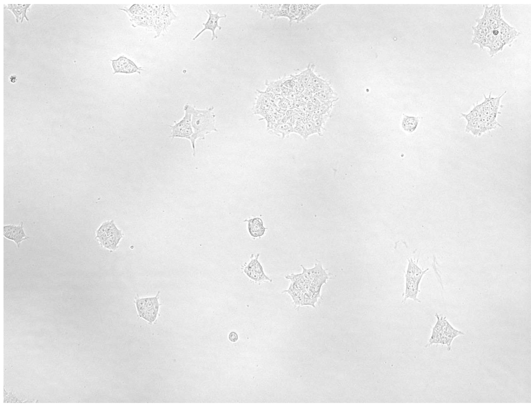	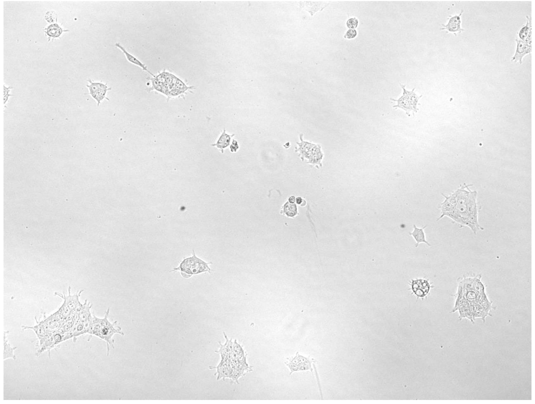
E8<br>lower<br>right	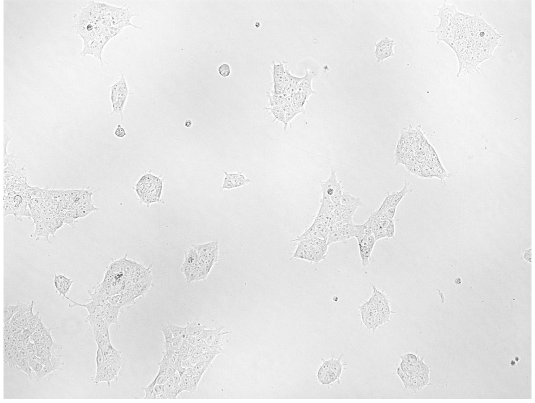	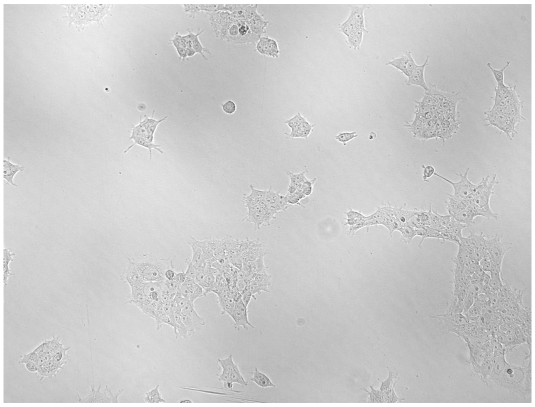

F8<br>right<br>middle	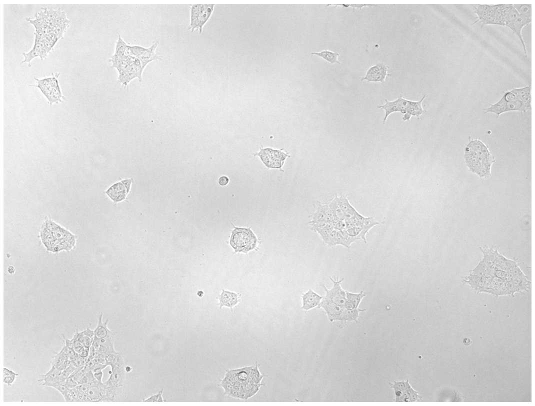	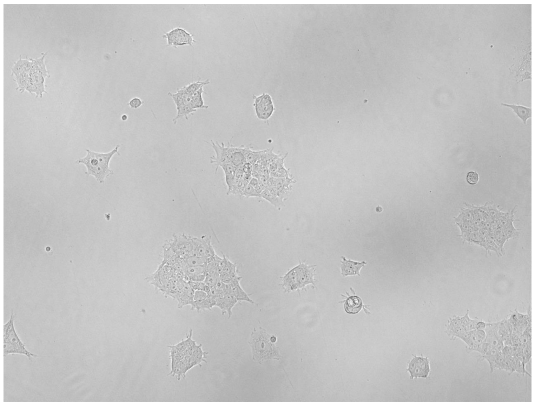
G3<br>center	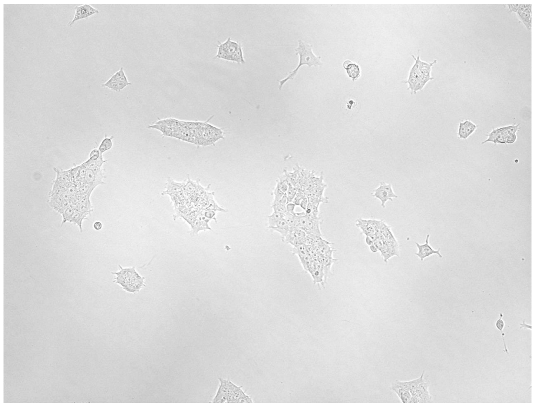	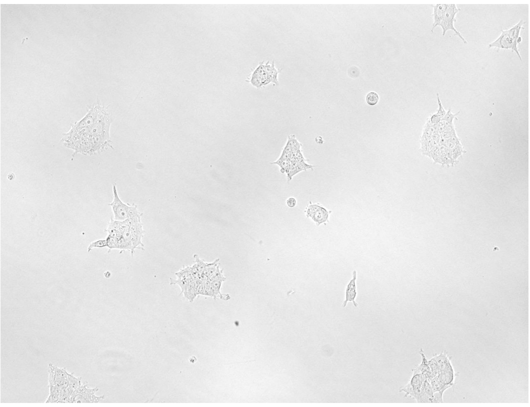
G4<br>upper<br>left	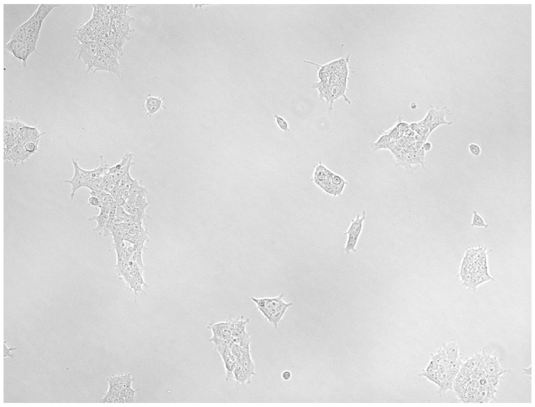	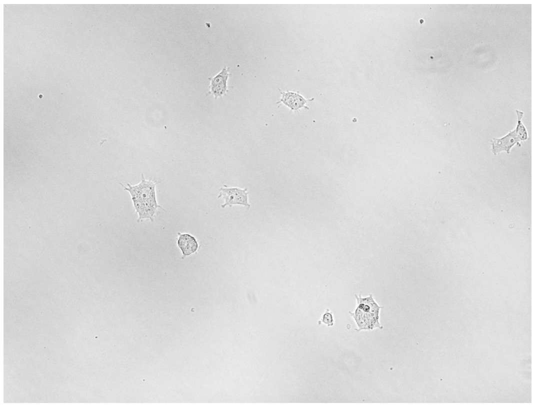

3-day-old plate

	Robot-split plate	Manually split plate

B2<br>upper<br>middle	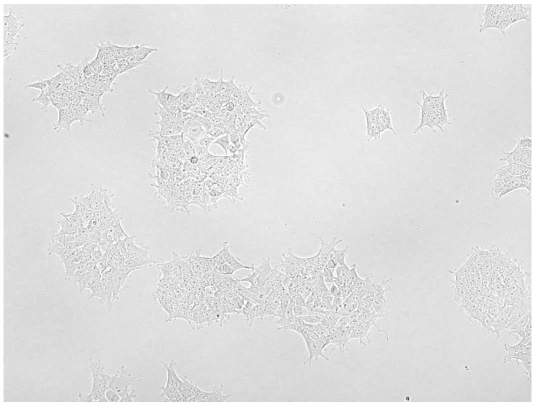	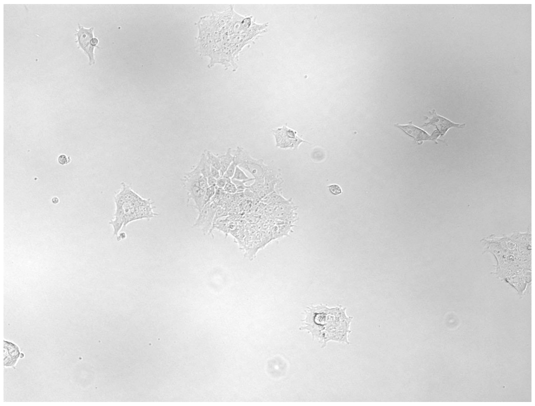
C9<br>lower<br>middle	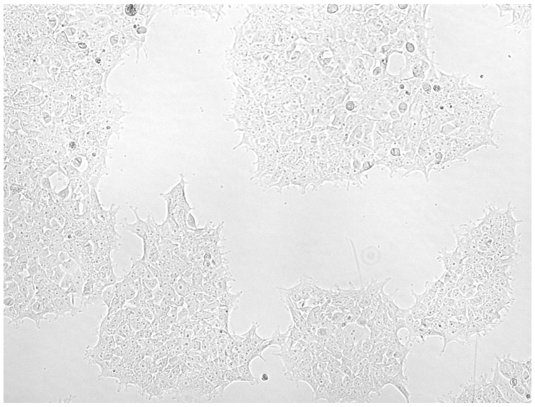	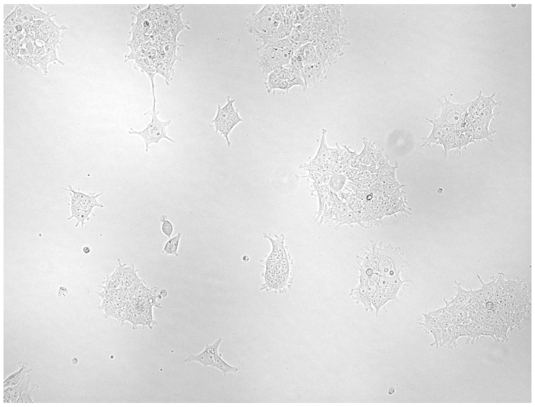
E8<br>lower<br>right	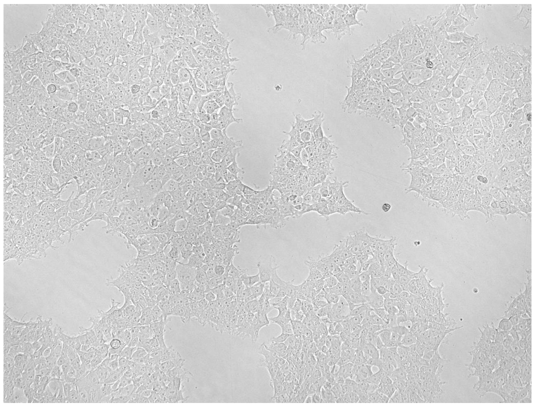	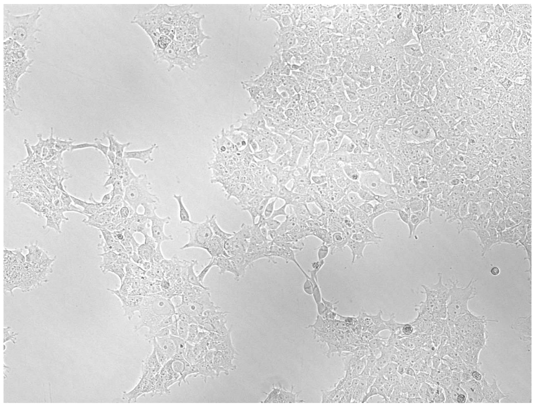

F8 right middle	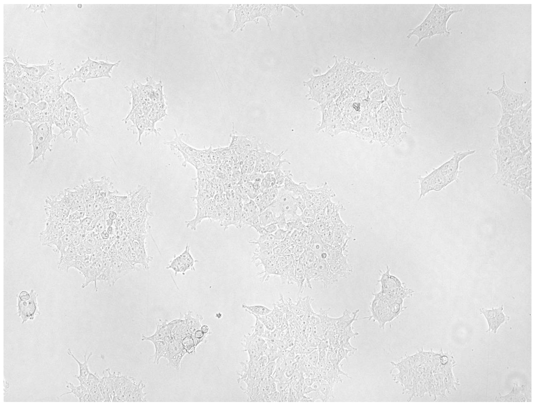	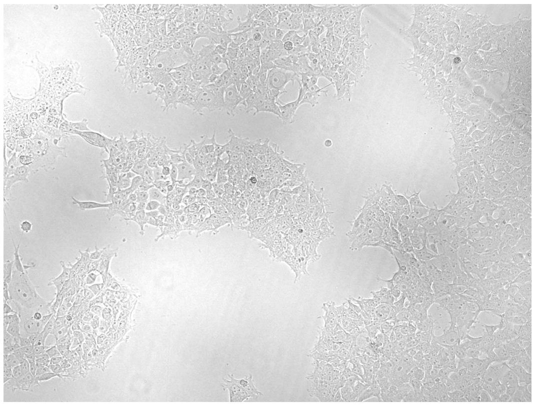
G3 center	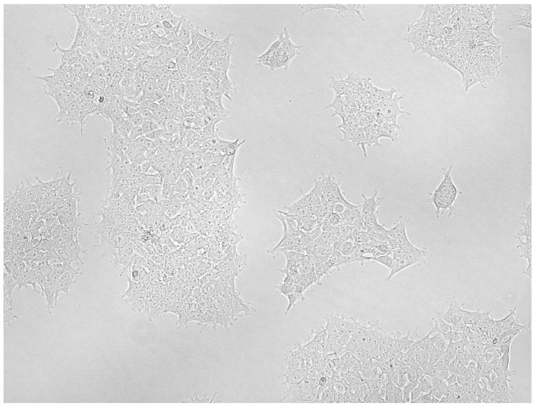	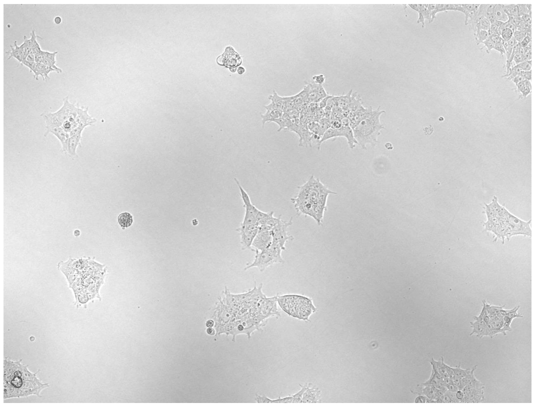
G4 upper left	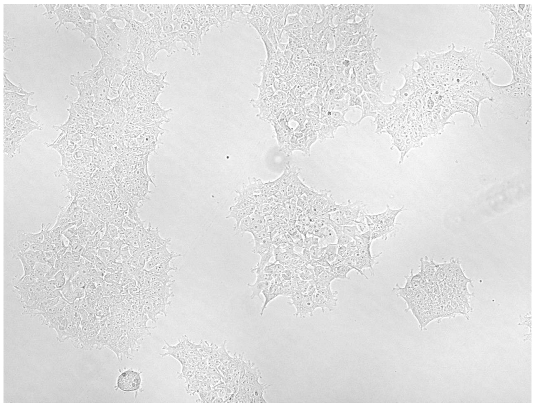	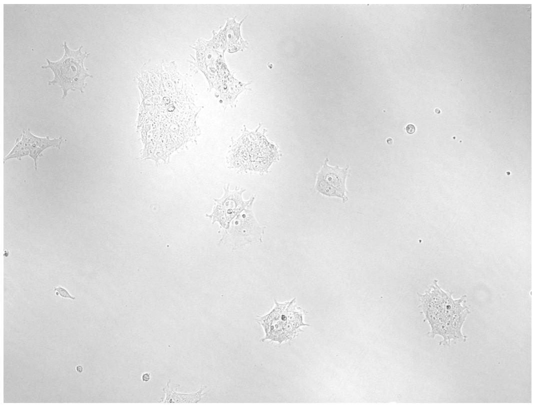

Materials and Methods

Reagents and cultures

Cells (HEK293T) and media used for this experiment are the same as described in the main text, Materials and Methods, subheading "Reagents and cultures".

Instruments

ACCS system, as described elsewhere in this publication, without Cell Counting Imager
Spectramax i3x plate reader with Minimax 300 Imaging Cytometer
EVOS FL digital microscope, using 10X objective
Invitrogen Countess II FL automated cell counter

Consumables

Corning 3610 white wall, clear bottom, TC-treated 96-well culture plates
Bio-Rad Microseal 'B' self-adhesive optical plate seals
Thermo Scientific #236272 white vinyl plate bottom seals
Invitrogen # C10228 Countess disposable counting slides

Test plates

On Monday, 6 source plates for the test plates are prepared by manually seeding 30k cells/well into all 96 wells of a Corning 3610 plate. Stocks for seeding are prepared by diluting a stock of freshly dissociated HEK293T cells according to a concentration measurement obtained with an Invitrogen Countess II cell counter.
On Tuesday, 6 test plates are made by passaging the source plates from Monday with a fixed split ratio of 1/6. Specific wells are left empty: C3, C7, F3, F7 for negative controls; columns 5 and 10 for reference wells.
3 of the source plates are seeded by hand according to the general manual tissue culture practices described in the main text ("Passaging of adherent HEK cells in 96 well plates") except that the dilution ratio is fixed, cell suspension is transferred by rows using a multichannel pipette, and wells are left empty as described above. 3 more source plates are seeded using an ACCS passaging protocol script designed to do the same thing but seeding one well at a time with the single-channel pipette. The protocol takes approximately 1.5 hours to run.

Viability assay

On each day Wednesday through Friday, one manually-seeded plate and one robot-seeded plate is assayed using CellTiter-Glo 2.0. An 11mL aliquot of thawed CellTiter-Glo 2.0 reagent and a 50mL tube of growth medium are moved from the refrigerator to ambient conditions on the morning of the measurement (at least 1 hour in advance) to allow them to equilibrate to room temperature. After imaging in the Spectramax is done, a white bottom seal is applied to the plate.

To prepare the plate: 100 µL of media is removed from all wells except for C3, C7, F3, F7. 100 µL of fresh media ("cells-" negative control) is added to C3 and F7 (round 2: F3 and C7). A stock is prepared from freshly dissociated cells, the concentration is measured with a Countess II cell counter, and dilutions are made at nominally 200k, 400k, 600k, and 800k cells/mL. Columns 5 and 10 are seeded with 100 µL of suspension each to create reference wells containing 80k, 60k, 40k, and 20k cells per well in a repeating pattern. Once all these additions are made to the plate, it is allowed to rest for 30 minutes to ensure it is in thermal equilibrium.

To initiate the assay, 100 µL of CellTiter-Glo 2.0 reagent is added to all wells except the two from which no media was removed ("reagent-" negative control). The lid is discarded and an optically clear top seal is applied. A Spectramax i3x plate reader is used to perform both the necessary mixing and the assay readout. The read is carried out in all-wavelength mode with a read height of 6.51mm and an integration time of 500 ms. The plate reader is configured to apply orbital shaking at the "high" intensity setting for 5 minutes (we have found that results are more inconsistent when shaking for only 2 minutes as suggested by the technical manual), then read the plate every 2 minutes for a total of 8 reads. In the analysis presented here we use the read around the 10 minute mark from the start of the run.

The 40k cells/well reference group was chosen as a scale ruler for the assay results as this cell density is well within the range over which CellTiter-Glo 2.0 is advertised to be highly linear. The raw luminance reads are zero-corrected with respect to that plate's "cells-" negative control group, then the resulting net values are scaled such that that plate's 40k cells/well reference group corresponds to a value of 1.0 in order to normalize for overall scale differences due to different source plate densities, etc.

Note that in practice, we find CellTiter-Glo 2.0 responds differently to a fresh cell suspension vs an established adherent culture, even with the increased mixing time applied in this round of measurements, so for this application we do not present the assay values as absolute cell counts.

Live imaging

The "round 2" plates were each imaged at 10X in phase contrast at 6 preselected sites just before being assayed. To reduce experimenter bias, the site selection was determined by random draw. Sites were defined by a 3x3 grid circumscribed by the border of each well; for each capture, the microscopist was directed to move the stage to place the FOV center inside the indicated square while viewing under 4X magnification, then switch to 10X and make only minimal position adjustments before taking the image.
In addition to the above, all 12 source plates and all 12 test plates were also imaged in their entirety in brightfield at 4X once per day for diagnostic purposes. 4X imaging was automated using a Spectramax i3x plate reader with MiniMax imaging cytometer. The instrument's chamber was set to 25°C when scanning plates headed for assays that day; for plates still in culture the temperature was set to 37°C.

A square circumscribed by the edge of the well bottom was divided into a grid of 9 squares, numbered 1-9 from left-to-right and top-to-bottom (i.e. 1 is upper left, 7 is lower left). 6 wells were pseudo-randomly selected, then for each well a grid location was selected. Two of the sites were arbitrarily changed due to favoring diversity in locations over true randomness. The technique for positioning the scope at locations other than #5 was to move to the corresponding side or corner of the well and then back towards the center until the well wall was off screen. For #5 the image would just be centered on the bright area in the middle of the well. After the initial position was acquired, the scope would be switched to 10X and phase illumination, focus was set for best contrast, and the image was taken. Minor repositioning was allowed for technical reasons, e.g. if too close to the opaque wall such that it occluded the illumination and reduced contrast. The goal was not to match exactly the same field of view every time, only to streamline the imaging task and reduce bias from unconscious "cherry picking" of wells/sites.

Data analysis

Data analysis and figure generation was performed with Python, Numpy and Matplotlib. See the Data Availability section below.
TIF images from the EVOS microscope were bulk converted to JPG format and individually given linear brightness and contrast adjustments for legibility. Raw images and unretouched JPEG versions can be found in the repository at the location mentioned in the Results section under "Morphology images."

Data availability

All supplemental materials, code, and documentation related to the ACCS publication can be found by starting at the main public repo:
https://github.com/czbiohub-sf/2024-accs-pub

Copies of images, protocol scripts, logs, and raw data to accompany this supplement can be found at:
https://github.com/czbiohub-sf/2024-accs-pub/accs24-pub-aux-data/supplement_e/
